# Real-world outcomes of atezolizumab–bevacizumab and durvalumab–tremelimumab in unresectable hepatocellular carcinoma: a systematic review with covariate-adjusted meta-regression and exploratory machine learning analysis

**DOI:** 10.3389/fimmu.2026.1956075

**Published:** 2026-09-24

**Authors:** Jongin Lee, Kwon Yong Tak, Hee Sun Cho, Jaejun Lee, Keungmo Yang, Pil Soo Sung, Jeong Won Jang, Ji Won Han

**Affiliations:** 1 Department of Occupational and Environmental Medicine, Seoul St. Mary’s Hospital, College of Medicine, The Catholic University of Korea, Seoul, Republic of Korea; 2 Division of Gastroenterology and Hepatology, Department of Internal Medicine, College of Medicine, The Catholic University of Korea, Seoul, Republic of Korea; 3 The Catholic University Liver Research Center, Department of Biomedicine & Health Sciences, College of Medicine, The Catholic University of Korea, Seoul, Republic of Korea

**Keywords:** comparative study, hepatocellular carcinoma, immune check inhibitor, machine learning, meta-analysis

## Abstract

**Background/purpose:**

Atezolizumab–bevacizumab (AB) and durvalumab–tremelimumab (DT) are standard first-line therapies for unresectable hepatocellular carcinoma (uHCC), but their comparative effectiveness remains uncertain.

**Methods:**

We included 76 observational reports comprising 10,243 patients. Report-level median survival was summarized descriptively, whereas objective response rate (ORR) and disease control rate (DCR) were synthesized using random-effects models. Covariate-adjusted meta-regression was the principal adjusted analysis, and adverse events were summarized descriptively. An exploratory prediction analysis using leakage-free nested cross-validation was performed, with an S-learner as the primary model and T-learners only as supporting analyses.

**Results:**

The evidence base comprised 60 AB reports (8,928 patients) and 16 DT reports (1,315 patients). For median overall survival (mOS), the sample size-weighted means were 17.7 months for AB and 16.5 months for DT; the corresponding medians of report-level medians were 17.1 and 13.8 months, respectively. These survival summaries were descriptive and were not formally compared. Random-effects pooled ORR was 26.6% for AB and 20.8% for DT, and pooled DCR was 71.4% and 55.4%, respectively. In covariate-adjusted meta-regression, the AB–DT differences were +3.57 months for mOS (95% CI, −0.38 to +7.52; P = 0.076), +1.96 months for median progression-free survival (mPFS) (+0.91 to +3.02; P < 0.001), +2.95 percentage points for ORR (−2.65 to +8.54; P = 0.302), and +16.15 points for DCR (+10.44 to +21.86; P < 0.001). The mPFS association was not reproduced in first line-predominant cohorts (+1.35 months, −0.33 to +3.04; P = 0.116) and attenuated after the simultaneous exclusion of four reports with unresolved overlap (+1.69 months, +0.31 to +3.07; P = 0.016).

**Conclusion:**

In available aggregate real-world data, AB cohorts reported higher median PFS and disease control than DT cohorts, whereas OS could not be reliably compared. Marked differences in treatment line, age, calendar period, and covariate support, together with the small DT evidence base, preclude reliable comparative-effectiveness inference.

**Systematic Review Registration:**

https://www.crd.york.ac.uk/PROSPERO/view/CRD420251141735, identifier CRD420251141735.

## Introduction

Hepatocellular carcinoma (HCC) remains a leading cause of cancer-related mortality worldwide, with the majority of patients diagnosed at an advanced stage where curative treatments are not feasible (1, 2). Recently, the landscape of systemic treatment for HCC has been revolutionized by the advent of immune checkpoint inhibitors (ICIs) (3). The landmark IMbrave150 phase III trial established atezolizumab plus bevacizumab (AB) as the preferred first-line therapy, demonstrating superior overall survival (OS) and progression-free survival (PFS) compared to sorafenib (4). Subsequently, the HIMALAYA phase III trial introduced the STRIDE regimen [single tremelimumab plus regular-interval durvalumab (DT)] as another first-line standard, also showing superior clinical outcomes to sorafenib (5).

Current international guidelines, including those from the American Association for the Study of Liver Diseases (AASLD) and the European Association for the Study of the Liver (EASL), recommend both AB and DT as first-line systemic therapies (6–8). While these guidelines provide high-level evidence for each regimen against sorafenib, they do not offer a preference between the two immunotherapies. Consequently, clinicians face a dilemma in selecting the optimal regimen, as there are no completed head-to-head randomized controlled trials (RCTs) directly comparing AB and DT (9). Although indirect comparisons using network meta-analysis (NMA) are theoretically possible, they are often hindered by the lack of a common comparator arm in single-arm real-world studies (RWSs) and substantial heterogeneity across study populations (10, 11). Since the recent introduction of the DT regimen, comparative real-world evidence (RWE) remains limited, and individual patient data (IPD) meta-analysis—although ideal—is currently logistically unfeasible across independent registries.

We therefore conducted a systematic review of real-world AB and DT cohorts to describe regimen-specific outcome distributions and to examine report-level adjusted associations. Median survival was treated as a descriptive outcome; formal random-effects synthesis was restricted to response proportions. Covariate-adjusted meta-regression was the principal adjusted analysis, with an exploratory report-level prediction analysis used only to assess the direction and stability of model-based contrasts. Because all comparisons rely on aggregate, predominantly single-arm observational data, none is interpreted as a causal regimen effect.

## Materials and methods

### Study design and registration

Reporting for this systematic review and meta-analysis was structured according to Preferred Reporting Items for Systematic Reviews and Meta-Analyses (PRISMA) (12). The protocol was prospectively registered in PROSPERO (CRD420251141735). The primary evidence synthesis comprised observational real-world reports of AB or DT in unresectable hepatocellular carcinoma (uHCC). Regimen-specific outcomes were described, and adjusted report-level associations were examined using meta-regression; prediction modeling was an explicitly exploratory extension.

### Search strategy

We searched PubMed, Embase, and the Cochrane Library from inception, using the complete database-specific queries reported in **Supplementary Table S1**. The search was executed on 27 August 2026, from inception and without date restriction. No study-design term was used as a mandatory search filter. Reference lists of relevant reviews and eligible reports were examined manually. A separate targeted search for reports directly comparing AB and DT within the same source population was also undertaken. Two of the nine comparator reports reached full-text assessment and appeared among the full-text exclusions in **Supplementary Figure S1**; the other seven were also retrieved by the corrected search, but were excluded at title-and-abstract screening as ineligible for the single-arm pool, and were assembled for tabulation by the targeted search. All nine reports were tabulated as contextual evidence and were not pooled with the primary single-arm evidence.

### Eligibility criteria

Eligible reports were required to describe a prospective or retrospective observational cohort of at least 10 adults with unresectable or advanced HCC treated with AB or DT in any treatment line and to provide at least one extractable efficacy outcome [median overall survival (mOS), median progression-free survival (mPFS), objective response rate (ORR), or disease control rate (DCR)]. Interventional trials, case reports, reviews, editorials, reports without quantitative outcomes, and duplicate reports of the same cohort were excluded. Directly comparative observational reports identified by the targeted search were retained as contextual evidence but were not pooled with the single-arm reports. Reports in which median survival was not reached, not estimable, or not reported did not contribute to that survival summary and were not imputed.

Two reviewers independently assessed possible cohort overlap by comparing authorship, institutions, enrollment periods, sample sizes, baseline characteristics, and reported outcomes. When reports represented the same cohort, the most complete report was retained. Reports with possible but unquantifiable partial overlap were retained in the primary analysis and examined via individual and simultaneous exclusion; a further sensitivity analysis retained one report per authorship-overlap family. Disagreements were resolved by consensus.

### Data extraction and quality assessment

Two investigators independently extracted study design, region, enrollment period, treatment line, median age, sex, viral etiology, Child–Pugh class, alpha-fetoprotein, Eastern Cooperative Oncology Group (ECOG) performance status, macrovascular invasion (MVI), extrahepatic spread (EHS), outcome definitions, response criteria, and endpoint-specific denominators. Efficacy outcomes were mOS, mPFS, ORR, and DCR. Any-grade and grade ≥3 or otherwise-defined severe adverse events were extracted where reported. No value was imputed for an unreported or not-reached median. Discrepancies were resolved by consensus. Methodological quality was assessed independently using the Newcastle–Ottawa Scale (NOS), classifying reports as high (≥7), moderate (5–6), or low (<5) quality (13).

### Primary analyses: descriptive synthesis and meta-regression

Report-level mOS and mPFS were summarized descriptively as the sample size-weighted mean of reported medians and the median of report-level medians with the interquartile range. Confidence intervals, prediction intervals, I², funnel plots, and Egger’s tests were not calculated for median survival because a valid report-specific standard error for a censored median could not be derived from sample size alone. ORR and DCR were synthesized using one-step DerSimonian–Laird random-effects models with binomial within-report standard errors (14). For the single report with zero ORR, p was set to 0.5/n only when calculating the standard error; the observed effect remained 0%. Heterogeneity for these proportions was summarized using I² (15). The principal adjusted analysis was a complete-case random-effects meta-regression of each outcome on regimen, later-line proportion, median age, and MVI prevalence. These coefficients were interpreted as ecological associations because aggregate-data adjustment cannot resolve patient-level confounding (16). Reports were weighted by 1/(SE² + τ²). The approximate within-report SE was median/(2√n) for mOS and mPFS, and √[p(1 − p)/n] for ORR and DCR, using the endpoint-specific denominator. The survival approximation was used only to weight the adjusted ecological meta-regression and was not treated as a valid standard error for censored-median inference. Because this approximation does not capture the uncertainty of a censored median, the survival meta-regressions are reported as exploratory ecological analyses, and specifications that do not use the approximation—sample size-weighted and unweighted least squares with HC3 robust standard errors—are reported alongside; residual I² is reported only for the proportion models. Residual I² was calculated as 100 × τ²/(τ² + s²), where s² is the typical within-report variance generalized to the residual degrees of freedom (16). A later-line proportion of 30% or less was adopted to define first line-predominant cohorts. Additional sensitivity analyses used alternative weights, leave-one-DT-report-out analyses, simultaneous exclusion of unresolved-overlap reports, one report per overlap family, and exclusion of reports using only the modified Response Evaluation Criteria in Solid Tumors (mRECIST). Funnel plots and Egger’s tests on the logit scale were restricted to ORR and DCR (17).

### Descriptive safety synthesis

Safety was summarized descriptively because adverse-event definitions and ascertainment varied across reports. For each adverse-event category, every report with a non-missing value in **Supplementary Table S2** contributed; percentages were weighted by the baseline sample size and presented with observed ranges. No formal comparative safety analysis was performed.

### Exploratory prediction analysis

As an exploratory extension, report-level prediction models were used to examine whether model-predicted regimen contrasts were directionally consistent with the meta-regression; related machine learning approaches have been used for prognostication and treatment selection in HCC (18–20). Complete-case datasets comprised 48 reports for mPFS (40 AB and 8 DT) and 45 for DCR (35 AB and 10 DT), with no imputation. Candidate algorithms were elastic net, ridge, least absolute shrinkage and selection operator (LASSO), support vector regression, random forest, and gradient boosting. Predictive performance was estimated using repeated, treatment-stratified fivefold outer cross-validation (six repeats), with hyperparameters tuned in an inner fourfold loop and all preprocessing confined to the training folds; pooled out-of-fold R² was compared with an intercept-only baseline. The elastic net model for mPFS and random forest model for DCR, as specified in the submitted methods, were retained. An S-learner provided the primary exploratory contrast by toggling the regimen indicator within a single fitted model. Separate-arm T-learners were retained only as supporting analyses (21). Ten covariate profiles were evaluated against marginal DT-arm support; six required extrapolation, and four lay within every marginal range. For each of 500 bootstrap replicates, reports were resampled independently within each treatment arm; the resulting intervals describe model variability rather than confidence intervals for a treatment effect, and no P or q values were assigned to model-predicted contrasts. Shapley additive explanation (SHAP) values were used only for qualitative model interpretation (22). Analyses used R 4.3.1 and Python 3.10; code is available at https://github.com/tmznjf/ML-based-meta-analysis_HCC-immunotherapy.

## Results

### Study selection and characteristics

The overall study outline is presented in **Figure 1**. The corrected primary review included 76 observational reports: 60 AB reports (8,928 patients) and 16 DT reports (1,315 patients), for a total of 10,243 patients (**Supplementary Figure S1**). Sixty-four reports were retrospective, and 12 were prospective; 52 (68.4%) originated from Asian settings and 24 from Western settings. Publication years ranged from 2021 to 2026. Median follow-up across reporting cohorts was 10.05 months (range, 4.0–48.2). Median OS, median PFS, ORR, and DCR were available in 45, 62, 60, and 56 reports, respectively. The mean later-line proportion was 19.6% in AB cohorts and 43.2% in DT cohorts; their corresponding medians were 5.2% and 54.8%. Among reports providing age, the mean of report-level median ages was 68.4 years for AB (range, 57.0–82.0; k = 59) and 71.7 years for DT (62.0–75.6; k = 15). These differences, together with calendar-time separation and limited covariate overlap, indicate potential confounding between regimen groups (**Supplementary Table S2**).

**Figure 1:**
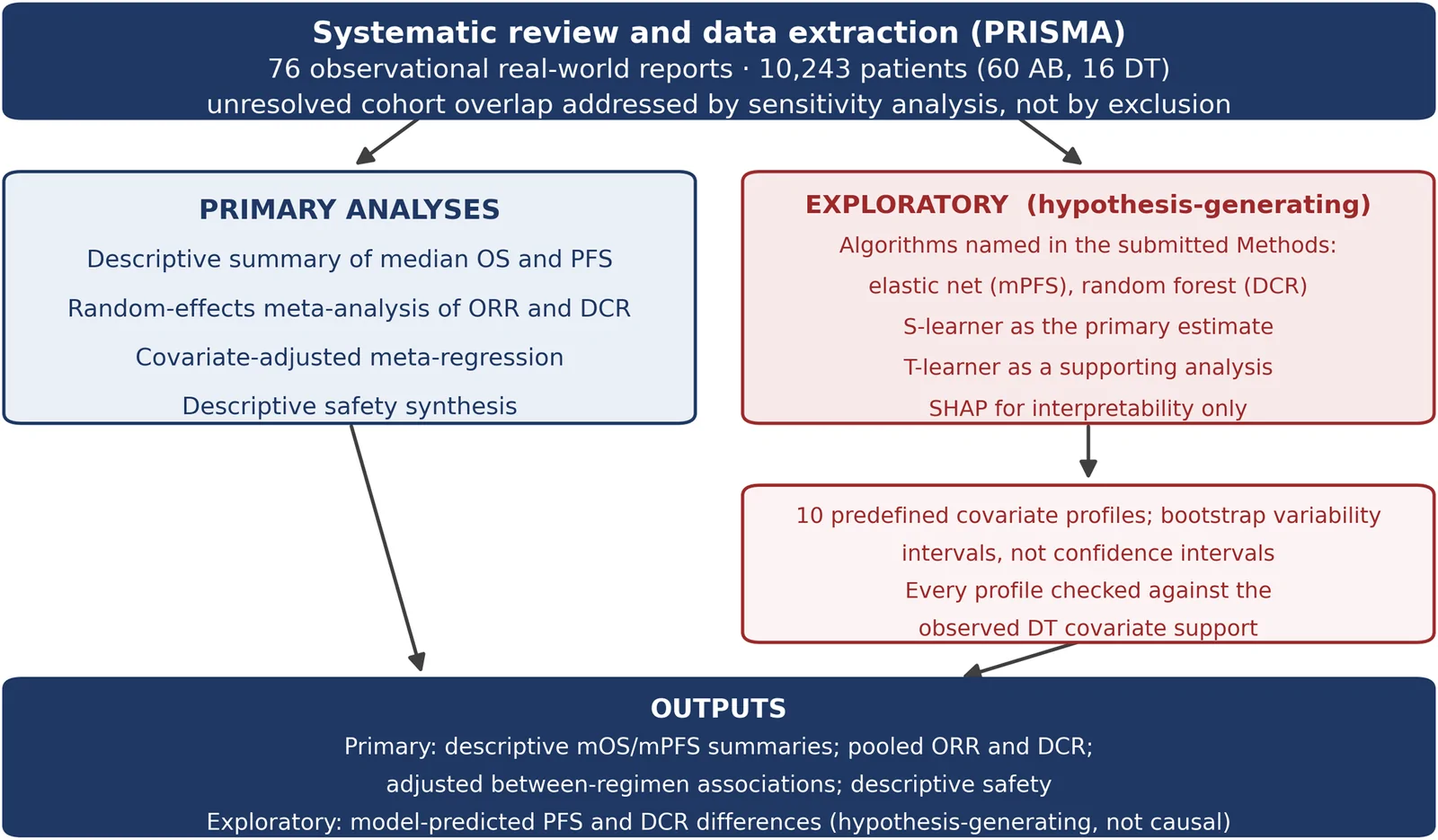
Evidence-selection and analysis flow.

Methodological quality was moderate in 19 reports (25.0%) and low in 57 (75.0%); none met the NOS threshold for high quality. The principal limitations were inadequate between-group comparability and incomplete outcome ascertainment in predominantly single-arm observational designs (**Supplementary Table S2**).

### Descriptive efficacy outcomes

For mOS, the sample size-weighted mean of report-level medians was 17.7 months for AB (39 reports; 6,993 patients) and 16.5 months for DT (6 reports; 732 patients); the corresponding medians of medians were 17.1 months [interquartile range (IQR), 12.8–20.0] and 13.8 months (10.7–17.5). For mPFS, the weighted means were 6.9 months for AB (50 reports; 7,617 patients) and 4.5 months for DT (12 reports; 1,030 patients), and the medians of medians were 6.6 months (IQR, 5.4–7.9) and 3.9 months (3.5–5.8). These survival summaries are descriptive and do not constitute formal between-regimen comparisons. Random-effects pooled ORR was 26.6% (95% CI, 23.8–29.4; I² = 84.0%) for AB and 20.8% (16.7–24.8; I² = 54.8%) for DT. Pooled DCR was 71.4% (68.8–74.0; I² = 77.3%) and 55.4% (52.5–58.4; I² = 0.0%), respectively (**Table 1**; **Figure 2**).

**Table 1:** Pooled real-world efficacy outcomes.

Endpoint	Regimen	Reports (n)	RWS N	Pooled real-world estimate	I²	Pivotal trial
mOS	AB	39	6,993	17.7 (weighted mean); 17.1 median of medians [IQR 12.8–20.0]	—	19.2 months
	DT	6	732	16.5 (weighted mean); 13.8 median of medians [IQR 10.7–17.5]	—	16.4 months
mPFS	AB	50	7,617	6.9 (weighted mean); 6.6 median of medians [IQR 5.4–7.9]	—	6.9 months
	DT	12	1,030	4.5 (weighted mean); 3.9 median of medians [IQR 3.5–5.8]	—	3.8 months
ORR	AB	46	6,711	26.6 [95% CI, 23.8–29.4]	84.0%	27.3%
	DT	14	1,048	20.8 [95% CI, 16.7–24.8]	54.8%	20.1%
DCR	AB	42	5,674	71.4 [95% CI, 68.8–74.0]	77.3%	73.6%
	DT	14	1,048	55.4 [95% CI, 52.5–58.4]	0.0%	60.1%

AB, atezolizumab–bevacizumab; DT, durvalumab–tremelimumab; mOS, median overall survival; mPFS, median progression-free survival; ORR, objective response rate; DCR, disease control rate; CI, confidence interval; IQR, interquartile range; RWS, real-world studies.

**Figure 2:**
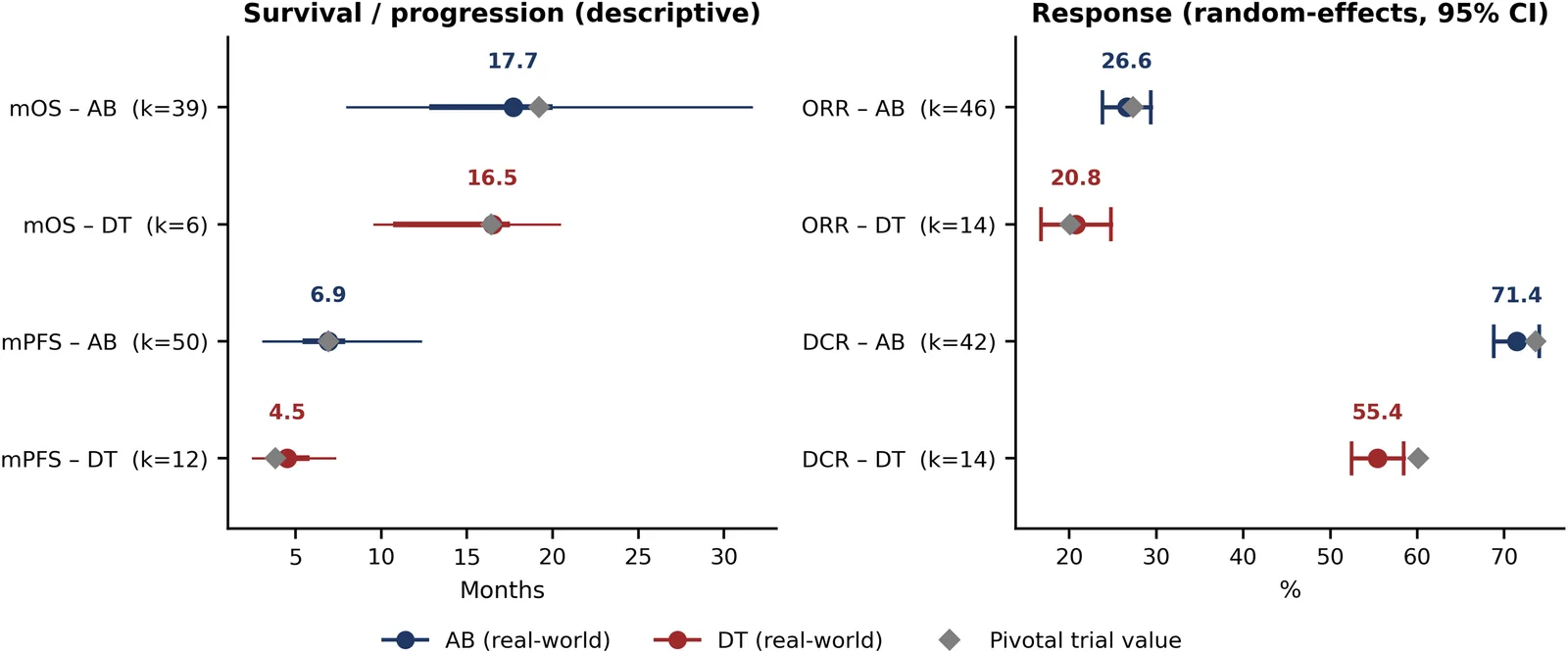
Pooled real-world outcomes compared with the pivotal trials. Left: median overall survival (mOS) and median progression-free survival (mPFS) are descriptive summaries—circle, sample size-weighted mean of report-level medians; thick bar, interquartile range; thin line, observed range; no confidence intervals are shown, as a valid report-specific standard error for a censored median could not be derived. Right: objective response rate (ORR) and disease control rate (DCR) from DerSimonian–Laird random-effects meta-analysis with 95% confidence intervals. Gray diamonds are corresponding pivotal-trial values, shown for context only; no formal indirect comparison was performed.

### Covariate-adjusted meta-regression

In the covariate-adjusted meta-regression (**Table 2**), the AB–DT coefficient was +3.57 months for mOS (95% CI, −0.38 to +7.52; P = 0.076; k = 37, including five DT reports), +1.96 months for mPFS (+0.91 to +3.02; P < 0.001; k = 52), +2.95 percentage points for ORR (−2.65 to +8.54; P = 0.302; k = 53), and +16.15 points for DCR (+10.44 to +21.86; P < 0.001; k = 49). In first line-predominant cohorts (later line ≤30%), the mPFS coefficient was attenuated and no longer statistically distinguishable from zero (+1.35 months, −0.33 to +3.04; P = 0.116; k = 30), whereas the DCR coefficient was +14.70 points (+5.12 to +24.29; P = 0.003; k = 22). Both analyses contained only four DT reports (**Supplementary Table S3**). Leave-one-DT-report-out estimates ranged from +1.67 to +2.20 months for mPFS and +14.57 to +17.05 points for DCR; after simultaneous exclusion of four reports with unresolved overlap, the coefficients were +1.69 months (+0.31 to +3.07; P = 0.016) and +14.78 points (+7.86 to +21.71; P < 0.001), respectively. Under specifications that do not use the survival variance approximation, the mPFS coefficient was +1.79 months (+0.37 to +3.22; P = 0.014) with sample size-weighted HC3 least squares and +1.98 months (+0.71 to +3.24; P = 0.002) unweighted, whereas the mOS coefficient remained inconclusive under both (+2.51 months, −6.49 to +11.50, P = 0.585; and +3.72 months, −0.63 to +8.07, P = 0.093) (**Supplementary Table S3**).

**Table 2:** Covariate-adjusted meta-regression of treatment (AB vs. DT).

Outcome (adjusted)	Difference, AB − DT (95% CI unless stated)	P	Reports (k)	AB/DT	Residual I²
mOS	+3.57 months (−0.38 to +7.52)	0.076	37	32/5	—
mPFS	+1.96 months (+0.91 to +3.02)	<0.001	52	43/9	—
ORR	+2.95 points (−2.65 to +8.54)	0.302	53	41/12	72.7%
DCR	+16.15 points (+10.44 to +21.86)	<0.001	49	37/12	64.3%
Sensitivity analyses					
mPFS, first line-predominant (later line ≤30%)	+1.35 (−0.33 to +3.04)	0.116	30	26/4	—
DCR, first line-predominant (later line ≤30%)	+14.70 (+5.12 to +24.29)	0.003	22	18/4	—
mPFS, leave-one-DT-report-out (range)	+1.67 to +2.20 months	max 0.005	51	—	—
DCR, leave-one-DT-report-out (range)	+14.57 to +17.05 points	max <0.001	48	—	—
mPFS, all unresolved-overlap reports removed	+1.69 months (+0.31 to +3.07)	0.016	48	—	—
DCR, all unresolved-overlap reports removed	+14.78 points (+7.86 to +21.71)	<0.001	45	—	—
Exploratory prediction analysis (S-learner, primary)—the interval is a bootstrap 95% variability interval, not a confidence interval, and no P-value is reported					
mPFS (elastic net)	+1.30 months (+0.46 to +2.20)	—	48	40/8	—
DCR (random forest)	+10.21 points (+0.85 to +17.18)	—	45	35/10	—

Model: outcome ~ treatment + later-line proportion + median age + macrovascular-invasion prevalence; random-effects meta-regression with the one-step DerSimonian–Laird between-report variance estimator and normal-approximation inference; complete-case analysis. Residual I² is reported only for the proportion models; for the survival models, it would rest on the median/(2√n) approximation and is therefore not reported.

AB, atezolizumab–bevacizumab; DT, durvalumab–tremelimumab; mOS, median overall survival; mPFS, median progression-free survival; ORR, objective response rate; DCR, disease control rate; CI, confidence interval.

Univariate report-level associations are reported in **Supplementary Tables S4, S5** and should be interpreted as ecological. Regimen-specific DT models contained only 5–14 reports, making individual coefficients unstable. Alternative weighting schemes and source-discrepancy analyses did not materially change the direction of the mPFS or DCR coefficients (**Supplementary Table S3**). Funnel plots were restricted to ORR and DCR (**Supplementary Figure S2**). On the logit scale, Egger’s tests suggested small-study effects for ORR in both arms and for AB DCR (all P < 0.001), but not for DT DCR (P = 0.944); given the small arm-specific report counts and clinical heterogeneity, these findings were not interpreted as proof of publication bias.

### Descriptive safety outcomes

Safety reporting remained heterogeneous. Any-grade adverse events were reported in 16 AB reports (2,259 patients; weighted mean 66.7%, range 26.7%–93.8%) and 5 DT reports (367 patients; 71.9%, 34.4%–76.8%). Grade ≥3 or otherwise-defined severe events were reported in 16 AB reports (2,337 patients; 31.2%, 10.0%–71.0%) and 7 DT reports (477 patients; 20.5%, 9.4%–36.6%). These values include every non-missing adverse-event percentage in **Supplementary Table S2** and are descriptive only (**Table 3**).

**Table 3:** Descriptive safety synthesis.

Adverse event	Regimen	Reports (n)	Patients (n)	Weighted mean % (range)
Any-grade	AB	16	2,259	66.7% (26.7–93.8)
Any-grade	DT	5	367	71.9% (34.4–76.8)
Grade ≥3/severe	AB	16	2,337	31.2% (10.0–71.0)
Grade ≥3/severe	DT	7	477	20.5% (9.4–36.6)

AB, atezolizumab–bevacizumab; DT, durvalumab–tremelimumab; AE, adverse event.

### Directly comparative real-world cohorts and exploratory prediction analysis

Nine real-world reports compared AB and DT within the same source population (**Supplementary Table S6**). Because serial reports arose from the same federated or multicenter networks, the nine reports represented only four independent source families. None showed a statistically significant difference in their principal overall-survival comparison, although one report described a significant 1-year landmark result. Three reports, arising from two source families, reported numerically higher DCR with AB, and two reported longer median PFS with AB; safety findings were mixed. These non-randomized comparisons were not pooled and are presented only as contextual evidence.

In leakage-free nested cross-validation, pooled out-of-fold R² was +0.114 for the elastic net mPFS model and +0.266 for the random forest DCR model, compared with intercept-only baselines of −0.041 and −0.034, respectively (**Supplementary Table S7**; **Figure 3A**). SHAP values (**Supplementary Figure S3**) indicated that the regimen indicator contributed no more to the mPFS model than MVI prevalence or viral etiology (mean |SHAP| 0.36, 0.35, and 0.35 months) and, although it ranked first in the DCR model (3.5 points), was followed by median age (2.4) and later-line proportion (1.1)—the covariates that differ most between the AB and DT cohorts—so that its contribution cannot be separated from cohort composition. The primary S-learner model-predicted contrast was +1.30 months for mPFS (bootstrap 95% variability interval, +0.46 to +2.20; 48 reports) and +10.21 percentage points for DCR (+0.85 to +17.18; 45 reports), in the same direction as, but approximately two-thirds the size of, the meta-regression coefficients (**Figure 3B**). Supporting T-learner predictions, fitted to only 8 DT reports for mPFS and 10 for DCR, favored AB in 45 of 48 and in all 45 reports, respectively; however, reports with above-median predicted mPFS benefit had a higher later-line proportion (36% vs. 12%) and older median age (71.5 vs. 66.7 years), indicating that predicted benefit tracked cohort composition rather than patient-level effect modification (**Supplementary Figures S4, S5**). Across the 10 covariate profiles, predicted differences ranged from +0.7 to +3.2 months and from +3.0 to +19.8 points (**Supplementary Tables S8, S9**; **Supplementary Figure S6**); six profiles required extrapolation beyond at least one marginal DT covariate range (**Supplementary Table S10**; **Supplementary Figure S7**), both extremes arose under extrapolation, and the four profiles within support yielded intermediate values close to the meta-regression estimates. Across the seven covariates, the observed DT range spanned only 19%–72% of the corresponding AB range (**Figure 3C**), so that even profiles within every marginal range could lie outside the region jointly occupied by the DT reports. The scenario estimates and SHAP rankings are therefore reported as qualitative descriptions of model behavior rather than as subgroup or treatment-effect estimates.

**Figure 3:**
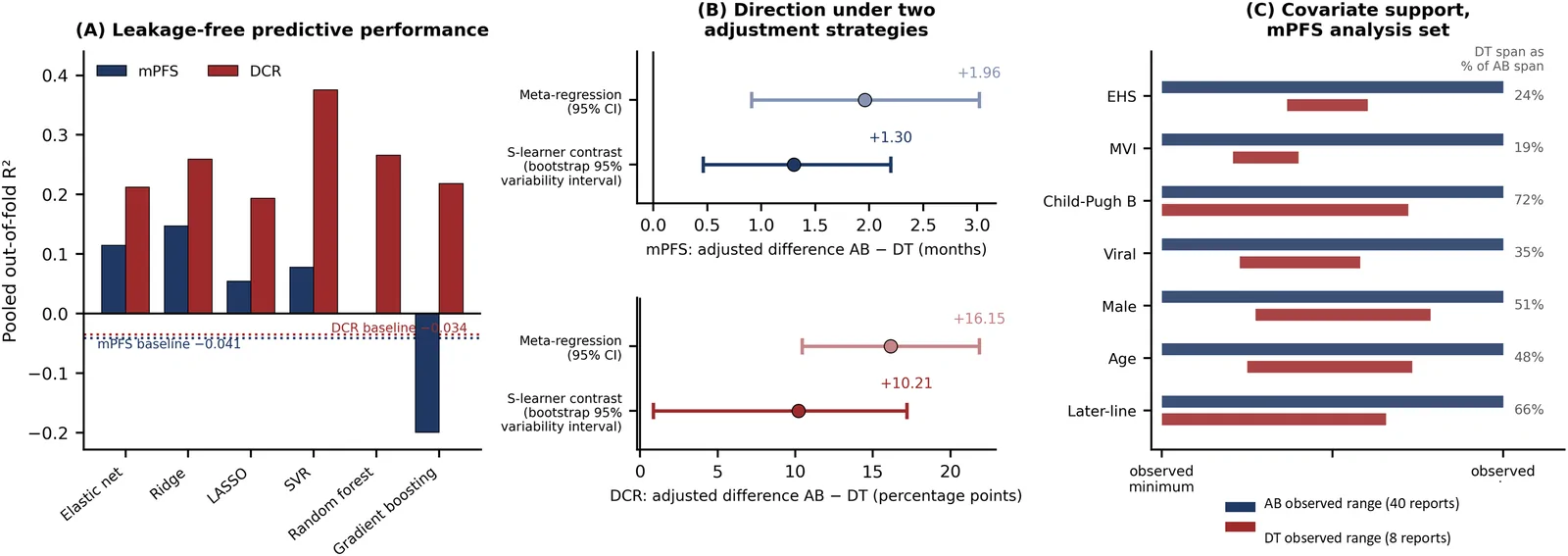
Exploratory report-level prediction analysis. **(A)** Pooled out-of-fold R² under leakage-free nested cross-validation for each candidate algorithm; dotted lines are the intercept-only baselines. **(B)** Adjusted difference from the covariate-adjusted meta-regression beside the S-learner contrast, plotted on separate axes for each endpoint. **(C)** Covariate support in the median progression-free survival (mPFS) analysis set: navy and red bars are the ranges observed within the atezolizumab–bevacizumab (AB) and durvalumab plus tremelimumab (DT) arms, each covariate scaled to its own observed range, with the DT span at the right as a percentage of the AB span.

## Discussion

This review synthesizes 76 observational reports comprising more than 10,000 patients and, to our knowledge, provides the first pooled synthesis of real-world DT outcomes (16 reports; 1,315 patients) alongside AB. The regimen-specific summaries were broadly compatible with the pivotal trials (4, 5), and the AB estimates were also compatible with prior real-world synthesis (23). This external similarity does not validate a between-regimen comparison, as the source cohorts differed materially in treatment line, age, calendar period, eligibility, and covariate support. Accordingly, the reported PFS and DCR differences are interpreted as report-level associations rather than evidence of regimen superiority.

The distinction between early endpoints and OS remains clinically relevant. The durable survival tail reported with STRIDE in the 5-year HIMALAYA follow-up (24) cannot be inferred from cross-study differences in median PFS or DCR. Conversely, higher reported DCR in AB cohorts does not establish a long-term survival advantage. The descriptive mOS evidence for DT was particularly sparse, resting on six reports, and the adjusted OS model included only five DT reports.

Confounding by indication is the principal limitation. The mean later-line proportion was 19.6% in AB cohorts and 43.2% in DT cohorts, with medians of 5.2% and 54.8%, respectively; DT cohorts were also older on average and accrued later in the treatment era. DT may preferentially be selected when bevacizumab is contraindicated because of varices, bleeding risk, or portal hypertension, variables that were not uniformly reported. The mPFS coefficient was not reproduced in first line-predominant cohorts and attenuated after simultaneous exclusion of unresolved-overlap reports. However, these sensitivities contained at most four DT reports and therefore do not estimate an unconfounded first-line comparison or demonstrate equivalence.

The 5-year HIMALAYA update confirms the standing of DT as a first-line standard (24), and current guidelines endorse both regimens without a head-to-head preference (7, 8, 25–27). The nine directly comparative real-world reports provide useful context but represent only four independent source families, include residual confounding, and, in one case, include durvalumab monotherapy in the comparator. Their principal OS comparisons were generally inconclusive, which is consistent with our decision not to infer comparative survival; the reported PFS and DCR directions remain hypotheses for prospective evaluation.

In addition to the durable survival tail noted above (24), the first-line landscape now includes nivolumab plus ipilimumab following CheckMate 9DW (28). These developments increase the need for direct comparative evidence. Apparent report-level modification by MVI or other tumor-burden variables should not be interpreted as patient-level effect modification. Mechanistic hypotheses concerning anti-VEGF vascular normalization (29) remain speculative in the absence of individual-level interaction analyses.

The exploratory prediction analysis did not overcome the disconnected, imbalanced evidence structure. Although the S-learner avoided fitting separate primary models to very small treatment arms, toggling the regimen indicator still required prediction across poorly overlapping covariate space. The T-learner was even less stable because the DT-specific models contained only 8 or 10 reports. Leakage-free cross-validation evaluated outcome prediction, not the validity of a regimen contrast. Directional agreement across models therefore indicates internal consistency only; it does not establish causal validity.

This study has several strengths. First, it assembles the real-world DT evidence base, which has not previously been synthesized, from a corrected search executed without date restriction and with independent dual screening of all newly retrieved records, and it additionally tabulates the nine directly comparative real-world cohorts identified, which represent four independent source families. Second, the analysis was designed to be checkable: endpoint-specific denominators, the between-report variance estimator, and the continuity convention are stated explicitly so that the pooled values can be recomputed from **Supplementary Table S2**; cohort overlap was audited by institution-level comparison and by a mechanical authorship screen, and its maximum influence was bounded by sensitivity analysis rather than assumed; and the prediction models were evaluated under leakage-free nested cross-validation with covariate support quantified for every profile. Third, by identifying where evidence is absent—first-line DT cohorts, DT survival, and joint covariate support—the review specifies the registries and comparative studies that are now required.

This study has several limitations. First, almost all evidence was observational and single-arm, with marked confounding by indication, limited overlap, and no common comparator. Second, the DT evidence base was small, particularly for mOS, first-line sensitivity analyses, and prediction models. Third, possible partial cohort overlap could not be quantified from publications, although individual, simultaneous, and family-level exclusions were examined. Fourth, key prognostic variables, response criteria, follow-up, and adverse-event definitions were incompletely and inconsistently reported; complete-case analysis may have introduced selection bias. Fifth, all reports were of low or moderate methodological quality. Sixth, medians of censored survival distributions could only be summarized descriptively. Seventh, funnel-plot asymmetry for proportions may reflect clinical heterogeneity and the mathematical relation between a proportion and its standard error rather than publication bias. Eighth, the first-line threshold was adopted during revision and left at most four DT reports. Ninth, the prediction results were unstable, frequently required extrapolation, and based on aggregate data.

In conclusion, available aggregate real-world data show that AB cohorts reported higher median PFS and DCR than DT cohorts, whereas OS could not be reliably compared. The PFS association was not robust to restriction to first line-predominant cohorts and attenuated after simultaneous exclusion of four reports with unresolved overlap. Marked between-cohort differences and the small DT evidence base preclude reliable comparative-effectiveness inference. The direction of the DCR findings is retained as a hypothesis for prospective head-to-head or individual-patient-data evaluation, not as evidence of a regimen effect.

## Data Availability

The original contributions presented in the study are included in the article/**Supplementary Material**. Further inquiries can be directed to the corresponding author.
