# Supplementary material for: Real-world outcomes of atezolizumab–bevacizumab and durvalumab–tremelimumab in unresectable hepatocellular carcinoma: a systematic review with covariate-adjusted meta-regression and exploratory machine learning analysis
Source: Front Immunol. 2026 Sep 24;17:1956075. doi: 10.3389/fimmu.2026.1956075 (PMC13646090; doi:10.3389/fimmu.2026.1956075)
Supplement: Supplementary file 1 [file Supplementaryfile1.pdf]

Figure S1

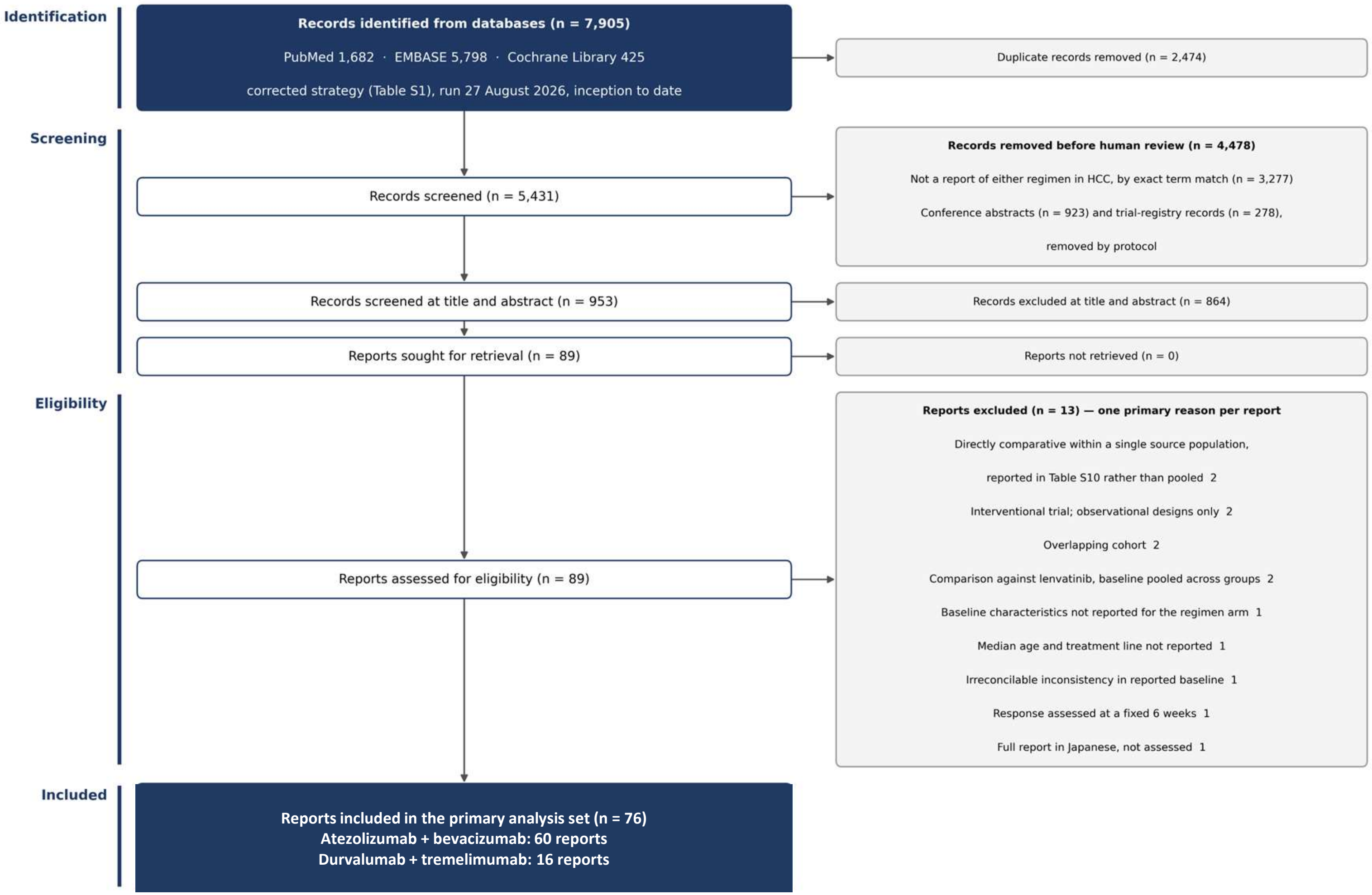

Supplementary Figure S1. PRISMA 2020 flow diagram.

Figure S2

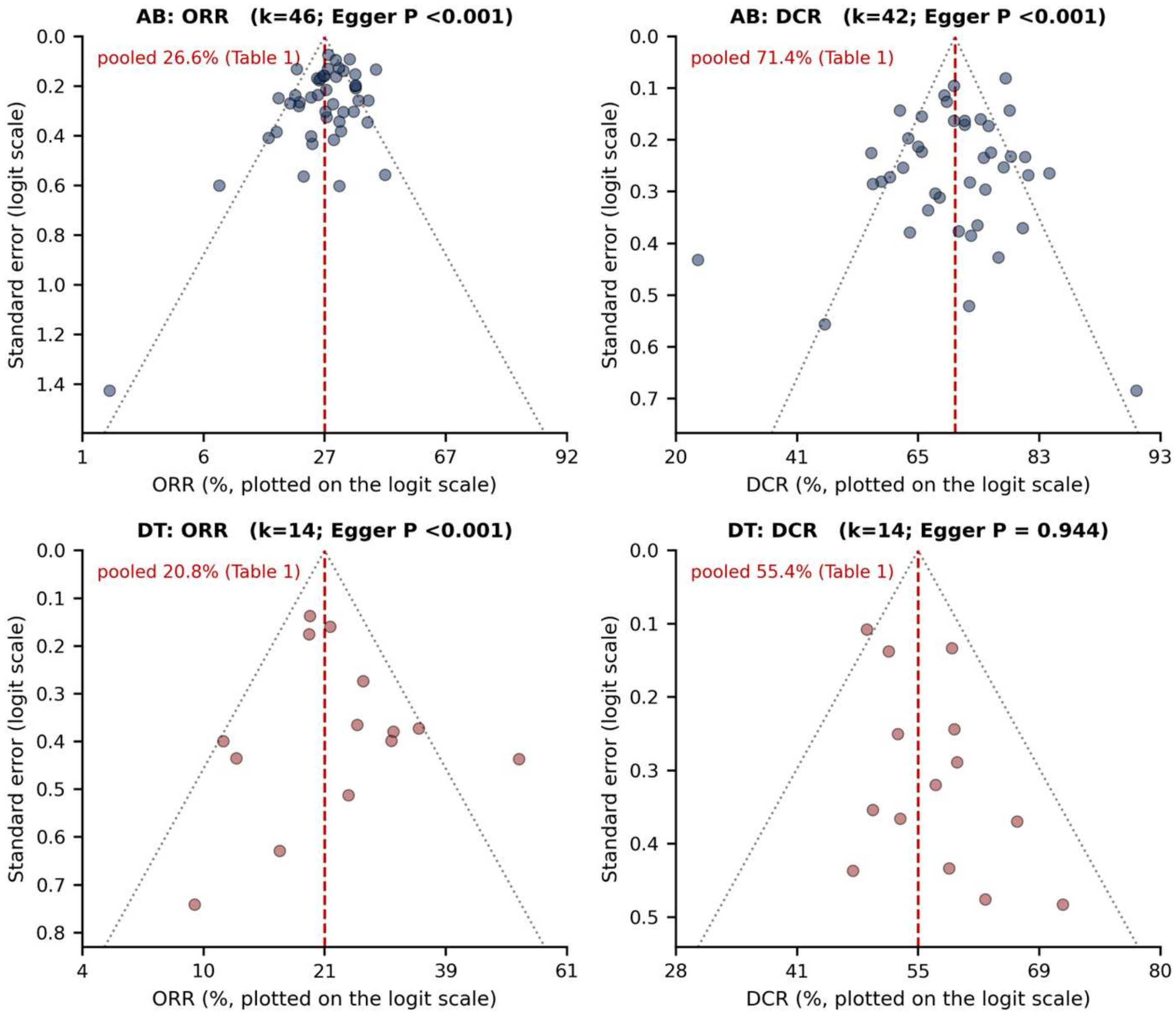

Supplementary Figure S2. Funnel plots for objective response rate and disease control rate. Funnel plots are shown only for proportions; those for median survival have been removed, because the pooled survival estimates are now descriptive. Plots are on the logit scale: for an untransformed proportion the standard error is a deterministic function of the estimate, which induces artefactual asymmetry. Vertical dashed lines are the pooled estimates; diagonal dotted lines are pseudo 95 % limits. Egger P values are shown in each panel.

Figure S3

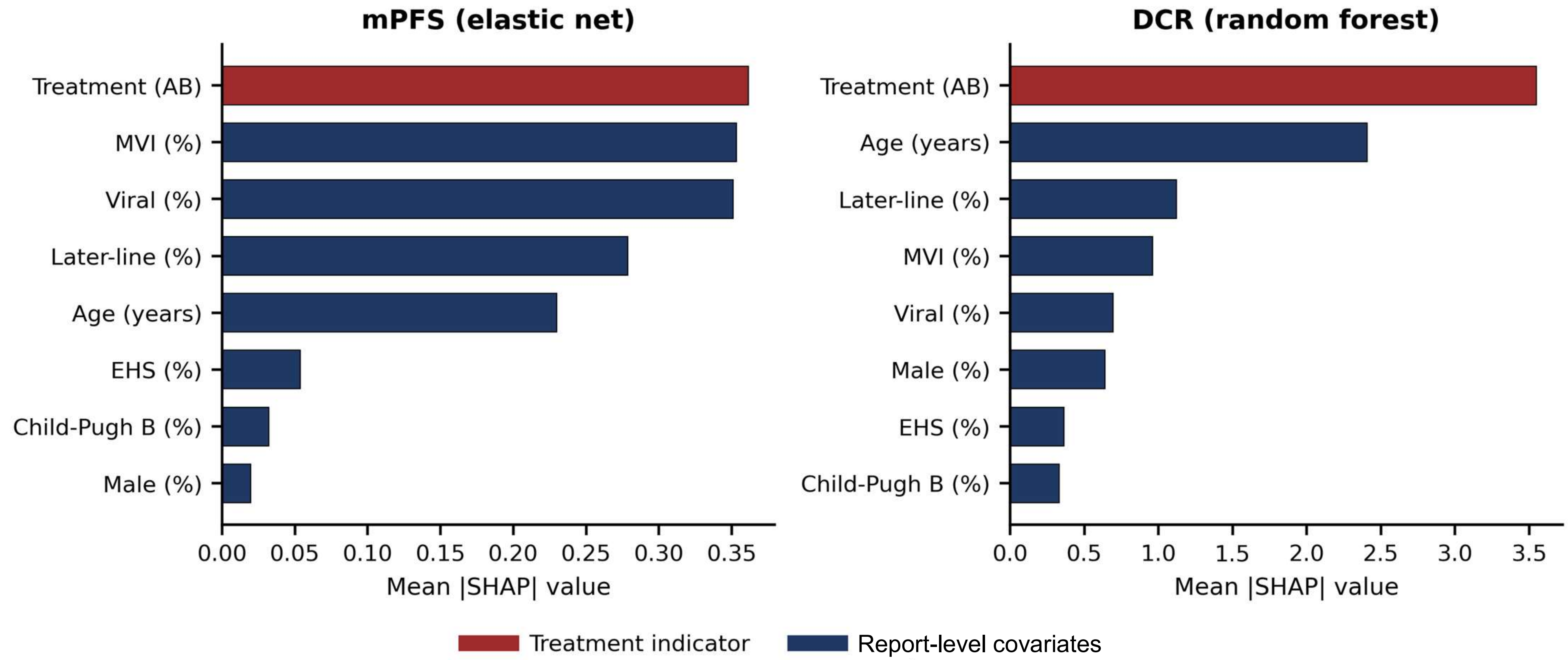

Supplementary Figure S3. SHAP feature importance for each endpoint (mPFS, elastic net, 48 reports; DCR, random forest, 45 reports), showing mean absolute SHAP values. Red, treatment indicator; navy, report-level covariates.

Figure S4

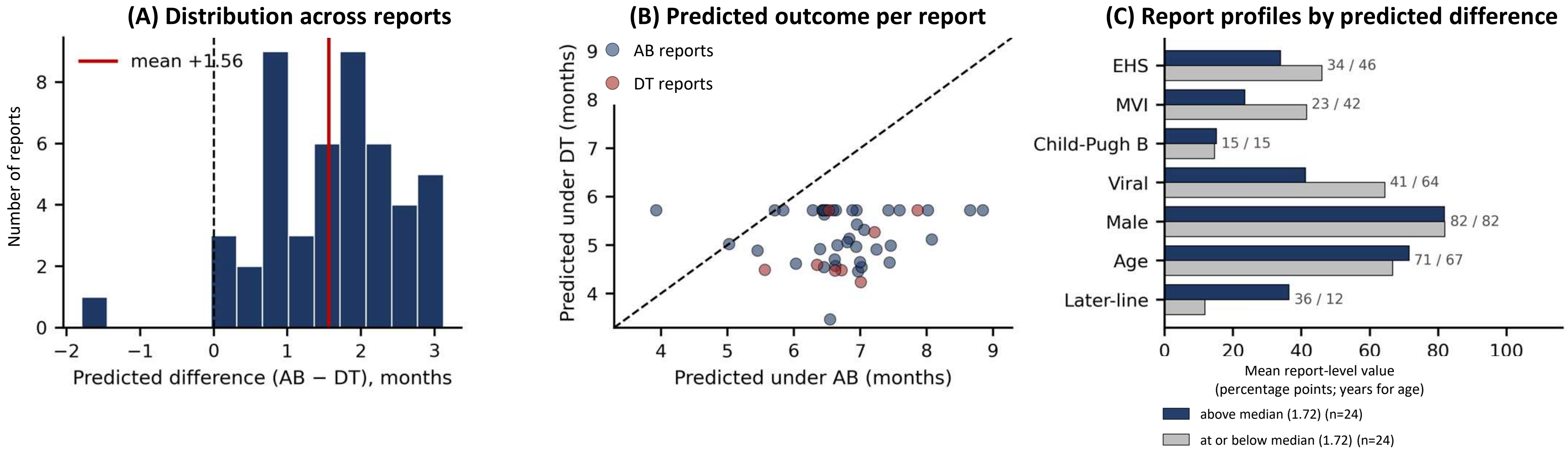

Supplementary Figure S4. Report-level model-predicted mPFS differences (T-learner; 48 reports, 40 AB and 8 DT). (A) Distribution across reports (mean +1.56 months). (B) Predicted mPFS under AB versus under DT for each report; the diagonal marks equality. (C) Mean report-level characteristics of reports grouped by whether the predicted difference exceeds the median predicted difference. Values are predictions from regimen-specific models evaluated at each report's covariate values, not individual-level treatment effects, and are ecological and hypothesis-generating only. Panel C is descriptive: the grouping is a presentational device for inspecting model behavior and defines no subgroup, and no comparison between the groups is tested.

Figure S5

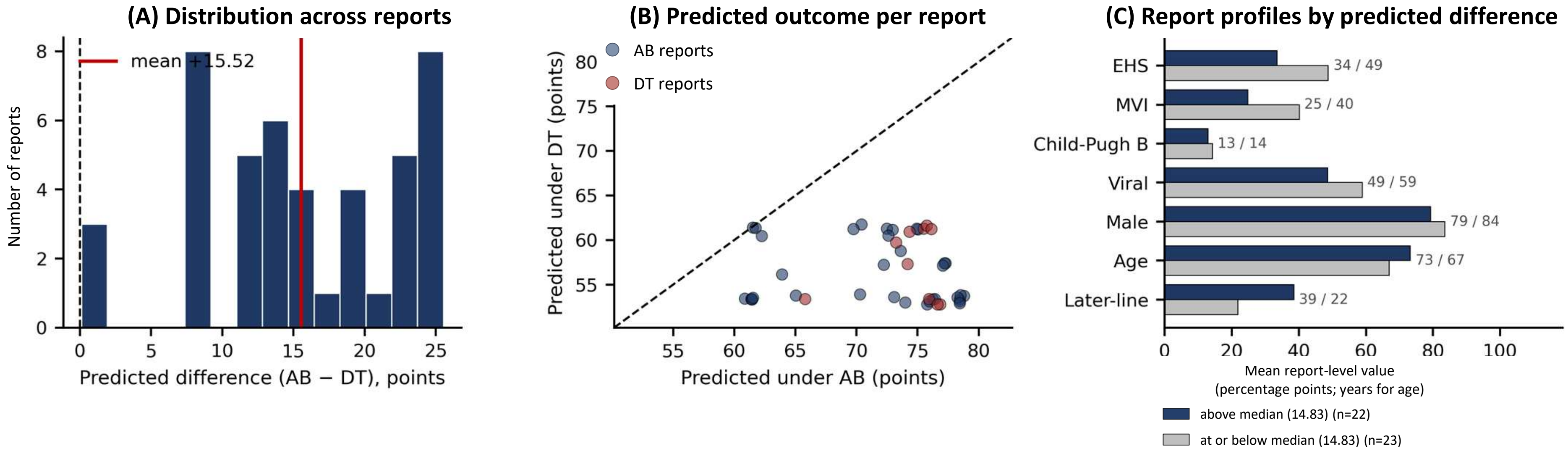

Supplementary Figure S5. Report-level model-predicted DCR differences (T-learner; 45 reports, 35 AB and 10 DT), presented as in Figure S4 with a grouping by t he median predicted difference in panel C.

Figure S6

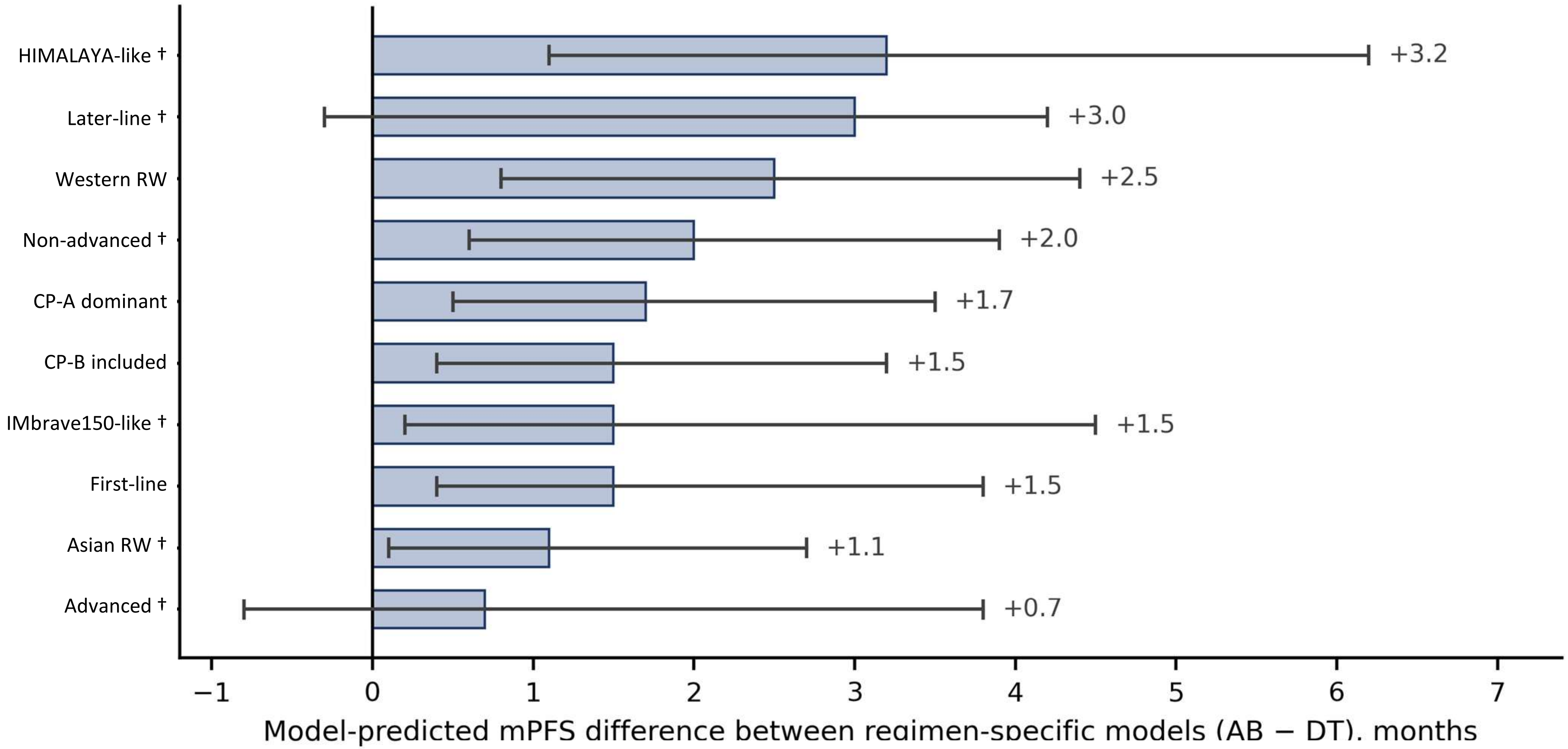

† Six profiles lie outside observed marginal DT support for at least one covariate: HIMALAYA-like, IMbrave150-like, Advanced, Non-advanced, Later-line, and Asian RW. No profile-level causal interpretation is warranted; DT sample size and joint covariate support remain limited.

Supplementary Figure S6. Exploratory scenario-level model-predicted mPFS differences (T-learner, supporting analysis). Each bar shows the difference between predictions from regimen-specific models evaluated at the corresponding covariate profile; they are not estimated treatment effects. Whiskers are bootstrap variability intervals, not confidence intervals for a treatment effect (500 resamples drawn independently within each arm). Daggers identify the six profiles requiring extrapolation beyond at least one marginal DT covariate range; four profiles lay within all marginal ranges.

Figure S7

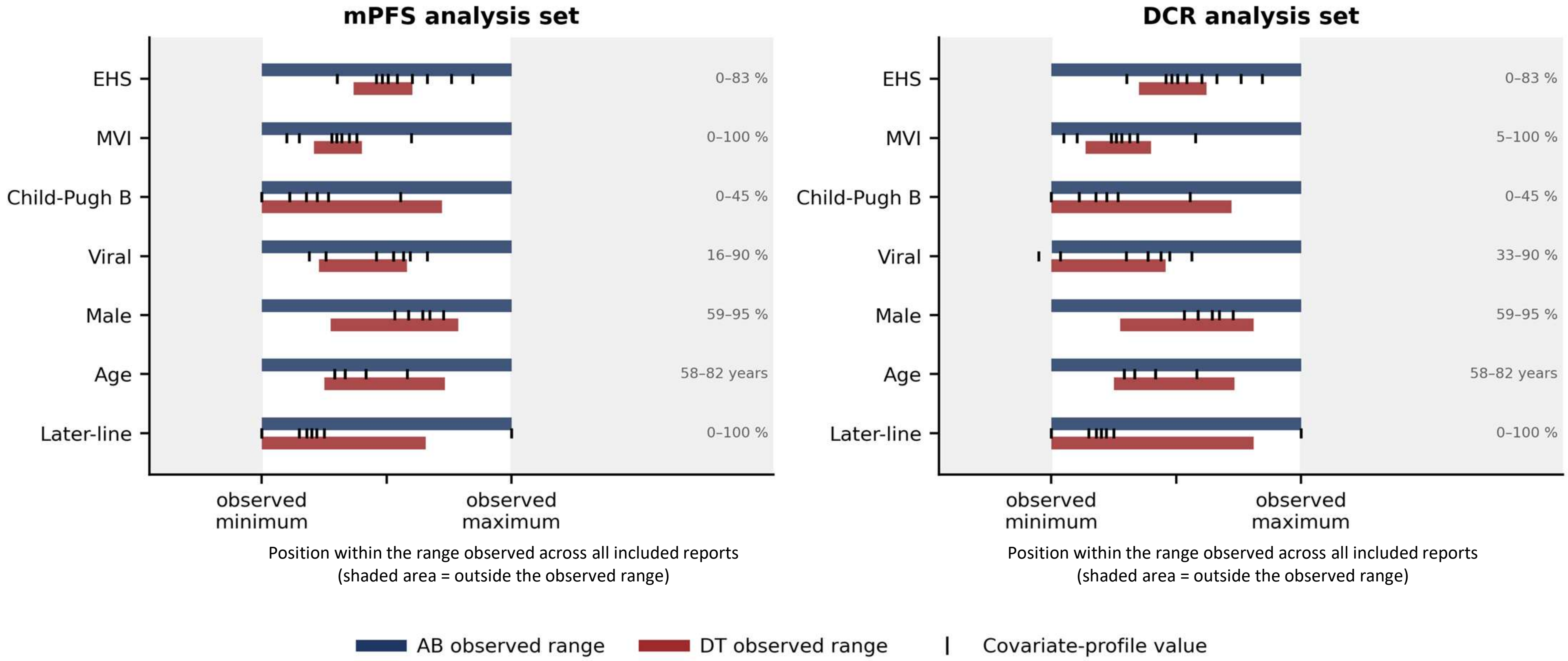

Supplementary Figure S7. Covariate support by treatment arm. Each covariate is scaled to its observed range within the corresponding analysis set, so that percentages and ages are not plotted on a shared numeric axis; the gray label at the right of each row gives that observed range in the original units. Navy and red bars are the ranges observed within the AB and DT arms; vertical ticks are the values specified by the ten covariate profiles.
