# Supplementary material for: Real-world outcomes of atezolizumab–bevacizumab and durvalumab–tremelimumab in unresectable hepatocellular carcinoma: a systematic review with covariate-adjusted meta-regression and exploratory machine learning analysis
Source: Front Immunol. 2026 Sep 24;17:1956075. doi: 10.3389/fimmu.2026.1956075 (PMC13646090; doi:10.3389/fimmu.2026.1956075)
Supplement: Supplementary file 2 [file Supplementaryfile2.docx]

**Table S1. Search strategy**

| **Database** | **Line** | **Query** | **Records** |
| --- | --- | --- | --- |
| PubMed | #1 | "Carcinoma, Hepatocellular"[Mesh] OR "Liver Neoplasms"[Mesh] OR "Carcinoma, Hepatocellular"[TW] OR "Hepatocellular Carcinoma"[TW] OR "Hepatocellular Carcinomas"[TW] OR "Liver Neoplasms"[TW] OR "Hepatic Neoplasm"[TW] OR "Hepatic Neoplasms"[TW] OR "Liver Cancer"[TW] OR "HCC"[TW] — P: population |  |
|  | #2 | "atezolizumab"[Supplementary Concept] OR "atezolizumab"[TW] OR "MPDL3280A"[TW] OR "MPDL-3280A"[TW] OR "Tecentriq"[TW] |  |
|  | #3 | "Bevacizumab"[Mesh] OR "Bevacizumab"[TW] OR "Avastin"[TW] OR "Mvasi"[TW] OR "Bevacizumab-awwb"[TW] |  |
|  | #4 | (#2 AND #3) OR "IMbrave150"[TW] OR "IMbrave 150"[TW] OR "atezolizumab plus bevacizumab"[TW] OR "atezolizumab-bevacizumab"[TW] — AB |  |
|  | #5 | "tremelimumab"[Supplementary Concept] OR "tremelimumab"[TW] OR "CP-675,206"[TW] OR "CP 675206"[TW] OR "CP-675206"[TW] OR "Imjudo"[TW] (NOTE: no generic monoclonal-antibody terms) |  |
|  | #6 | "durvalumab"[Supplementary Concept] OR "durvalumab"[TW] OR "MEDI4736"[TW] OR "MEDI-4736"[TW] OR "Imfinzi"[TW] (NOTE: no generic monoclonal-antibody terms) |  |
|  | #7 | (#5 AND #6) OR "HIMALAYA"[TW] OR "STRIDE"[TW] OR "durvalumab plus tremelimumab"[TW] OR "tremelimumab plus durvalumab"[TW] OR "durvalumab-tremelimumab"[TW] OR "tremelimumab-durvalumab"[TW] — DT |  |
|  | #8 | #1 AND (#4 OR #7) — FINAL. No first-line or systemic-therapy filter, no date limit | 1,682 |
| EMBASE | #1 | 'liver cell carcinoma'/exp OR 'liver tumor'/exp |  |
|  | #2 | ('hepatocellular carcinoma' OR 'liver cancer' OR 'liver neoplasm' OR 'hepatic neoplasm' OR 'HCC'):ti,ab,kw,de |  |
|  | #3 | #1 OR #2 — P: population |  |
|  | #4 | 'atezolizumab'/exp OR ('atezolizumab' OR 'MPDL3280A' OR 'Tecentriq'):ti,ab,kw,de |  |
|  | #5 | 'bevacizumab'/exp OR ('bevacizumab' OR 'Avastin' OR 'Mvasi'):ti,ab,kw,de |  |
|  | #6 | (#4 AND #5) OR ('IMbrave150' OR 'atezolizumab plus bevacizumab'):ti,ab,kw,de — AB |  |
|  | #7 | 'tremelimumab'/exp OR ('tremelimumab' OR 'CP 675206' OR 'Imjudo'):ti,ab,kw,de (NOTE: 'monoclonal antibody'/exp removed) |  |
|  | #8 | 'durvalumab'/exp OR ('durvalumab' OR 'MEDI 4736' OR 'Imfinzi'):ti,ab,kw,de (NOTE: 'monoclonal antibody'/exp removed) |  |
|  | #9 | (#7 AND #8) OR ('HIMALAYA' OR 'STRIDE' OR 'tremelimumab plus durvalumab' OR 'durvalumab plus tremelimumab'):ti,ab,kw,de — DT |  |
|  | #10 | #3 AND (#6 OR #9) — FINAL. No first-line or systemic-therapy filter, no date limit | 5,798 |
| Cochrane Library | #1 | [mh "Carcinoma, Hepatocellular"] OR [mh "Liver Neoplasms"] | 4,818 |
|  | #2 | (hepatocellular carcinoma OR HCC OR liver cancer OR liver neoplasm*):ti,ab,kw | 20,990 |
|  | #3 | #1 OR #2 | 20,991 |
|  | #4 | ((atezolizumab AND bevacizumab) OR IMbrave150):ti,ab,kw | 585 |
|  | #5 | ((tremelimumab AND durvalumab) OR HIMALAYA OR STRIDE):ti,ab,kw | 2,542 |
|  | #6 | #3 AND (#4 OR #5) — FINAL | 425 |

**Table S2. Characteristics of included reports**

| **Regimen** | **Report** | **Region** | **n** | **Design** | **Follow-up (mo)** | **Later-line (%)** | **Age (years)** | **Male (%)** | **Viral (%)** | **Child-Pugh B (%)** | **AFP (ng/mL)** | **ECOG 0 (%)** | **MVI (%)** | **EHS (%)** | **mOS (months)** | **mPFS (months)** | **ORR (%)** | **DCR (%)** | **NOS** | **Quality** | **n (survival)** | **n (response)** | **Response criteria** | **Enrollment period** | **Any-grade AE (%)** | **Grade ≥3 AE (%)** |
| --- | --- | --- | --- | --- | --- | --- | --- | --- | --- | --- | --- | --- | --- | --- | --- | --- | --- | --- | --- | --- | --- | --- | --- | --- | --- | --- |
| AB | Dhampalwar 2023 (1) | Western | 13 | prospective | — | 23.1 | 57 | 92.3 | 76.9 | — | — | 38.5 | 84.6 | 92.3 | 8 | — | 46.1 | — | 4 | Low | 13 | 13 | RECIST 1.1 | Not reported | — | — |
| AB | Yiu 2023 (2) | Asian | 13 | retrospective | — | 30.8 | 64 | 84.6 | — | 38.5 | — | — | 46.2 | 53.8 | — | 9.3 | 30.8 | 46.2 | 3 | Low | 13 | 13 | RECIST 1.1 | Not reported | — | — |
| AB | Nakabori 2024 (3) | Asian | 19 | retrospective | 25 | 0 | 75 | 94.7 | 36.8 | 5.2 | 57 | — | 5.3 | 57.9 | 19.8 | 7.4 | 21.1 | 73.7 | 4 | Low | 19 | 19 | RECIST 1.1 | 2020-11 to 2024-01 | 78.9 | 36.8 |
| AB | Kimura 2024 (4) | Asian | 21 | retrospective | 15.4 | 0 | 74.5 | 73.5 | 23.8 | 0 | 12.5 | 100 | 0 | 0 | 19.8 | 10.2 | — | — | 4 | Low | 21 | 21 | mRECIST | 2018-05 to 2023-10 | — | — |
| AB | **Ballester 2024 (5)** | Western | 23 | prospective | 10 | 13 | 70 | 91 | 48 | 17 | 12.5 | 43 | 30 | 44 | 12.7 | 4.7 | — | — | 4 | Low | 23 | 23 | mRECIST | 2020-12 to 2023-08 | — | — |
| AB | Tanaka 2022 (6) | Asian | 28 | retrospective | — | 17.9 | 73.5 | 79 | 46.4 | 0 | 310 | 89 | 25 | 32.1 | — | 10.5 | 29.2 | 91.7 | 4 | Low | 28 | 28 | RECIST 1.1 + mRECIST | 2021-01 to 2022-04 | — | — |
| AB | Charonpongsuntorn 2022 (7) | Asian | 30 | prospective | 10.1 | 0 | 58 | 90 | 73.3 | 0 | — | 6.7 | 56.7 | 56.7 | 10.2 | 6.7 | 23.3 | 63.3 | 3 | Low | 30 | 30 | RECIST 1.1 | 2020-09 onwards (end not stated) | — | 20.0 |
| AB | Park 2024 (8) | Asian | 30 | retrospective | 6.4 | 0 | 59.8 | 80 | 90 | 0 | 1349 | — | 100 | 53.3 | 10.8 | 3.6 | 0 | 23.3 | 4 | Low | 30 | 30 | RECIST 1.1 | 2020-10 to 2023-04 | 26.7 | 16.7 |
| AB | Matsumoto 2022 (9) | Asian | 32 | retrospective | 10.4 | 0 | 77 | 59 | 59 | 6 | 182 | 56 | 12 | 12 | 10.4 | 6.5 | 31.3 | 78.1 | 4 | Low | 32 | 32 | RECIST 1.1 | 2020-10 to 2022-04 | — | — |
| AB | Lee 2023 (10) | Asian | 35 | retrospective | — | 31 | 61 | 89 | 80 | 17 | — | 29 | 69 | 51 | 22.2 | 5.2 | 23 | 72 | 3 | Low | 35 | 35 | RECIST 1.1 | 2019-06 to 2022-01 | — | — |
| AB | Takada 2022 (11) | **Asian** | 35 | prospective | 9 | 49 | 73 | 83 | 69 | 25.7 | 36 | — | 23 | 29 | — | 5.8 | 40 | 74 | 3 | Low | 35 | 35 | RECIST 1.1 | 2020-11 onwards (end not stated) | — | — |
| AB | Kimura 2024 (12) | Asian | 36 | retrospective | — | 0 | 73 | 61.1 | 50 | 0 | — | 80.6 | — | — | 17.4 | — | — | — | 4 | Low | 36 | 36 | Not reported | 2018-01 to 2023-09 | — | — |
| AB | Mohamed 2022 (13) | **Western** | 37 | prospective | 18.8 | — | 70 | 83.8 | — | 21.6 | — | 81 | — | 35.1 | 12.2 | 3.1 | — | — | 5 | Moderate | 37 | 37 | Not reported | 2018-06 onwards (end not stated) | — | — |
| AB | Ando 2021 (14) | Asian | 40 | retrospective | 4 | 60 | 69 | 75 | 65 | 0 | 79 | — | 30 | 40 | — | — | 7.5 | 75 | 4 | Low | 40 | 40 | RECIST 1.1 + mRECIST | 2020-09 to 2021-07 | — | — |
| AB | Eso 2021 (15) | Asian | 40 | prospective | 6.9 | 35 | 70.5 | 87.5 | 47.5 | 5 | 19 | — | — | — | — | 5 | 30.8 | 66.7 | 4 | Low | 40 | 40 | RECIST 1.1 | 2020-10 onwards (end not stated) | — | — |
| AB | Su 2022 (16) | Asian | 46 | retrospective | 9.4 | 0 | 61.2 | 78.3 | 85.9 | 12 | — | 39.1 | 52.2 | 32.7 | — | 5.3 | — | — | 3 | Low | 46 | 46 | mRECIST | 2018-05 to 2022-05 | — | — |
| AB | Wang 2022 (17) | Asian | 48 | retrospective | 9.5 | 44.8 | 62 | 79.2 | 85.4 | 12.5 | 157.1 | 64.6 | 54.2 | 54.2 | — | — | 27.1 | 68.8 | 3 | Low | 48 | 48 | RECIST 1.1 | 2020-01 onwards (end not stated) | 93.8 | — |
| AB | Toshida 2023 (18) | Asian | 48 | retrospective | — | 52.1 | 82 | 81.3 | 39.6 | 18.7 | 17.3 | — | 18.7 | 37.5 | 25.6 | 7.5 | 35.4 | 81.3 | 5 | Moderate | 48 | 48 | RECIST 1.1 + mRECIST | 2018-04 to 2023-01 | — | — |
| AB | Sinner 2022 (19) | Western | 50 | retrospective | 10.1 | 48 | 65 | 82 | 33 | 22 | — | 54 | 34 | 54 | 31.6 | 7.1 | 32 | 68 | 2 | Low | 50 | 50 | RECIST 1.1 | 2018-12 to 2022-03 | — | — |
| AB | Hayakawa 2021 (20) | Asian | 52 | retrospective | 7.4 | 55.8 | 73 | 80.8 | 57.7 | 7.7 | — | 57.7 | 11.5 | 32.7 | — | 4.7 | 15.4 | 57.7 | 4 | Low | 52 | 52 | RECIST 1.1 | 2020-10 to 2021-07 | — | — |
| AB | Shigefuku 2024 (21) | Asian | 56 | retrospective | 14 | 0 | 74.3 | 80.4 | 32.1 | 7.1 | 48.5 | — | — | — | 20 | 7.5 | 26.8 | 59.4 | 4 | Low | 56 | 56 | RECIST 1.1 | 2018-04 to 2024-03 | — | — |
| AB | Sasaki 2024 (22) | Asian | 63 | prospective | 12.6 | 32 | 73 | 76.1 | 42 | 3.2 | 141 | 92 | 37 | 43 | — | 3.8 | 40.3 | 76.2 | 4 | Low | 63 | 63 | RECIST 1.1 | 2020-11 onwards (end not stated) | — | — |
| AB | Akyildiz 2023 (23) | Western | 65 | retrospective | 11.3 | 0 | 65 | 83.1 | 46.1 | 10.8 | 80 | 58.5 | — | 55.4 | 18.1 | 5.1 | 36.9 | 73.8 | 4 | Low | 65 | 65 | RECIST 1.1 | 2020-09 to 2023-03 | 29.2 | 10.7 |
| AB | **Himmelsbach 2022 (24)** | Western | 66 | retrospective | 7 | 0 | 65 | 82 | 34.8 | 34.8 | 17.6 | — | — | 27.3 | — | 6.5 | 29 | 62 | 4 | Low | 66 | 66 | RECIST 1.1 + mRECIST | 2018-12 to 2021-09 | 77.0 | 59.0 |
| AB | Kuo 2025 (25) | Asian | 80 | retrospective | 48 | 0 | 61.6 | 76.3 | 76.3 | 14.1 | 8802 | — | 55 | 50 | 10.4 | 3.7 | 20 | 55.7 | 3 | Low | 80 | 80 | RECIST 1.1 | 2019-12 to 2023-12 | — | — |
| AB | Hsu 2024 (26) | Asian | 84 | retrospective | 10.1 | 0 | 62.6 | 78.5 | 81.8 | 11.6 | 463 | — | 28.6 | 40.5 | 14.6 | 8.9 | — | — | 4 | Low | 84 | 84 | mRECIST | 2019-06 to 2023-08 | 73.8 | 19.0 |
| AB | Teng 2022 (27) | Asian | 89 | retrospective | 7.2 | 37.1 | 61.3 | 84.3 | 88.9 | 11.2 | 354 | 39.3 | 50.6 | 39.3 | — | 5.7 | 20.2 | 65.5 | 4 | Low | 89 | 89 | RECIST 1.1 + mRECIST | 2020-09 to 2022-03 | 61.0 | 10.0 |
| AB | Hu 2024 (28) | Asian | 92 | retrospective | 13.7 | 0 | — | 89.1 | 88 | — | — | — | 69.6 | 35.9 | — | 9.1 | — | — | 4 | Low | 92 | 92 | mRECIST | 2019-01 to 2023-06 | — | — |
| AB | Chuma 2021 (29) | Asian | 94 | retrospective | 4.8 | 46.8 | 73 | 77.7 | 52.1 | 13.8 | 105.6 | 77.7 | 23.4 | 34 | — | — | 18.1 | 78.8 | 4 | Low | 94 | 94 | RECIST 1.1 + mRECIST | 2020-10 to 2021-06 | 71.0 | 42.0 |
| AB | Suzuki 2024 (30) | Asian | 94 | retrospective | 17 | 26 | 73 | 79 | 41 | 15 | 18.9 | 94 | 31 | 48 | — | 7.4 | 23 | 82 | 4 | Low | 94 | 94 | RECIST 1.1 | 2020-10 to 2023-12 | — | — |
| AB | Jost-Brinkmann 2023 (31) | Western | 100 | retrospective | — | 4 | 67 | 87 | 42 | 25 | 88 | 69 | 54 | 38 | 20.3 | 6.3 | 36 | 76 | 4 | Low | 100 | 100 | RECIST 1.1 | 2020-01 to 2022-12 | 89.0 | 64.0 |
| AB | Kinami 2023 (32) | Asian | 109 | retrospective | 12.1 | 35.8 | 71 | 82.6 | 47.7 | 10.1 | 24.6 | — | 19.3 | 34.9 | 21 | 9.5 | 35.8 | 84.4 | 4 | Low | 109 | 109 | RECIST 1.1 + mRECIST | 2020-10 to 2022-11 | — | — |
| AB | Ledenko 2023 (33) | Western | 111 | retrospective | 8.1 | 6.3 | 68.4 | 80.2 | 47.7 | — | — | — | — | — | 12.9 | — | — | — | 4 | Low | 111 | 111 | Not reported | 2020-02 to 2022-08 | — | — |
| AB | **Song 2024 (34)** | Asian | 111 | retrospective | 5.1 | 0 | 61 | 78.4 | 78.4 | 8.1 | 55.7 | — | 37.8 | 82.9 | — | 6.5 | 27 | 63 | 4 | Low | 111 | 111 | RECIST 1.1 + mRECIST | 2022-05 to 2023-06 | 36.9 | — |
| AB | Lee 2024 (35) | **Western** | 112 | retrospective | 10.4 | 0 | 66 | 87 | 61 | 9 | 47 | 47 | 41 | 37 | 20.3 | 8 | 36 | 77 | 4 | Low | 112 | 112 | RECIST 1.1 | 2020-07 to 2022-08 | — | — |
| AB | **Sho 2022 (36)** | **Asian** | 115 | retrospective | 6.8 | 52.2 | 72 | 82.6 | 48.7 | 7.8 | 74.2 | 80 | 20 | 40 | — | 6.6 | 19.2 | 79.8 | 4 | Low | 115 | 115 | RECIST 1.1 + mRECIST | 2020-10 to 2022-01 | — | — |
| AB | **Nakagawa 2022 (37)** | Asian | 123 | retrospective | 7.6 | 51.2 | 73 | 82.9 | 49.6 | 7.3 | — | — | 27.6 | 41.5 | 11 | 5.9 | 15.8 | 81.6 | 4 | Low | 123 | 123 | RECIST 1.1 | 2020-10 to 2021-10 | — | — |
| AB | Singh 2025 (38) | Western | 160 | retrospective | 36 | 45 | 61.9 | 88 | 35.1 | 37.5 | — | — | 58.7 | 16.8 | 10 | 8 | — | — | 5 | Moderate | 160 | 160 | mRECIST | 2021-01 to 2023-12 | 53.0 | 20.0 |
| AB | Niizeki 2022 (39) | Asian | 161 | retrospective | 12.1 | 0 | 73 | 76.4 | 52.8 | — | 28.3 | — | 21.7 | 29.2 | — | 8.3 | — | — | 5 | Moderate | 161 | 161 | mRECIST | 2018-03 to 2022-09 | — | — |
| AB | D'alessio 2022 (40) | Western | 174 | retrospective | 9 | 0 | 69 | 85 | 53 | 24 | — | 63 | 40 | 77 | 14.9 | 6.8 | 25 | 73 | 4 | Low | 174 | 174 | RECIST 1.1 | 2019-01 to 2022-01 | 47.0 | 12.0 |
| AB | Takeuchi 2023 (41) | Asian | 184 | retrospective | — | 35.7 | 74 | 84.1 | 50 | 45 | 43.7 | 79.1 | 24.9 | 30.9 | — | 4.9 | 29.9 | 71.2 | 4 | Low | 184 | 184 | RECIST 1.1 | 2020 to 2022 | — | — |
| AB | Moriguchi 2024 (42) | Asian | 185 | retrospective | 17.6 | 36.8 | 75 | 84.9 | 42.2 | 38.4 | 19.1 | — | 15.7 | 32.4 | — | — | 25.4 | 65.5 | 5 | Moderate | 185 | 185 | RECIST 1.1 | 2020-09 to 2023-02 | — | — |
| AB | Kikuchi 2024 (43) | Asian | 188 | retrospective | — | 0 | 76 | 73.9 | 45.2 | 0 | 30.3 | 80.9 | 29.8 | 26.1 | — | — | 35.8 | 76.7 | 4 | Low | 188 | 188 | RECIST 1.1 | 2020-10 to 2022-09 | — | — |
| AB | Vithayathil 2023 (44) | Western | 191 | retrospective | — | 0 | 68.4 | 84.3 | 57.1 | 23 | — | 63 | 40.8 | 37.7 | — | — | 24.5 | 73 | 5 | Moderate | 191 | 191 | RECIST 1.1 | Not reported | 66.5 | 20.4 |
| AB | Castro 2024 (45) | Western | 207 | retrospective | 12 | 0 | 67 | 81.5 | 34.7 | 21.7 | 48 | 43 | 34 | 0 | 13 | 6.6 | 26.6 | 61.4 | 4 | Low | 207 | 207 | RECIST 1.1 | 2017 to 2024-04 | — | 71.0 |
| AB | Kaneko 2024 (46) | Asian | 213 | retrospective | — | 32.4 | 74 | 85.5 | 42.3 | 15.5 | 24.3 | 79.3 | 11.7 | 24.4 | 19.6 | 6.9 | 26.2 | 75.5 | 5 | Moderate | 213 | 213 | RECIST 1.1 | 2020-11 onwards (end not stated) | — | — |
| AB | Kim 2024 (47) | Asian | 246 | retrospective | 15.5 | 0 | 61 | 85 | 72.8 | 14.6 | — | 41.5 | 41.5 | 63 | 15.8 | 6.9 | 32.1 | — | 4 | Low | 246 | 246 | RECIST 1.1 | 2020-05 to 2023-05 | — | — |
| AB | Ueno 2024 (48) | Asian | 302 | retrospective | 11.8 | 29.1 | 73 | 78.8 | 50 | 41.7 | 19 | 74.2 | 17.9 | 33.1 | 23 | 9.5 | 27.5 | 79.6 | 5 | Moderate | 302 | 302 | RECIST 1.1 | 2020-10 to 2023-08 | — | — |
| AB | Kim 2025 (49) | Asian | 368 | retrospective | 11.9 | 0 | 61 | 81.5 | 74.7 | 0 | 77.8 | 34.9 | 44.6 | 67.9 | 18.5 | 6.3 | 19.6 | 69.6 | 4 | Low | 368 | 368 | RECIST 1.1 | 2013-10 to 2022-10 | — | — |
| AB | Cosgrove 2025 (50) | Western | 374 | retrospective | 5.6 | 0 | 68.8 | 78.9 | 51.3 | 27.5 | — | 39 | 31.6 | 31 | 13.2 | 6.4 | — | — | 5 | Moderate | 374 | 374 | Not reported | 2019-01 to 2022-11 | — | — |
| AB | Lee 2025 (51) | Asian | 374 | retrospective | 5.6 | 0 | 64.4 | 85.3 | 67.9 | 26.7 | — | 56.4 | 37 | 52.7 | 12.4 | 5.5 | — | — | 5 | Moderate | 374 | 374 | Not reported | 2020-09 to 2023-12 | — | — |
| AB | Kaplan 2024 (52) | Western | 405 | retrospective | 8.5 | 0 | 69 | 98 | 66 | 13.8 | — | — | — | 28.1 | 12.8 | — | — | — | 5 | Moderate | 405 | 405 | Not reported | 2017-01 to 2022-12 | — | — |
| AB | Tada 2024 (53) | Asian | 936 | retrospective | 12.8 | 33.5 | 74 | 79.1 | 49.3 | 10 | 30.7 | 83.5 | 20 | 31.2 | 22.3 | 6.5 | 27.6 | 79.1 | 5 | Moderate | 936 | 936 | RECIST 1.1 | Not reported | — | — |
| AB | Abdelrahim 2025 (54) | Western | 300 | retrospective | 8.7 | 0.0 | 68 | 79.3 | 44.0 | 26.3 | — | 36.7 | 25.3 | 40.0 | 14.4 | 6.8 | 30.7 | 70.0 | 4 | Low | 300 | 300 | RECIST 1.1 | 2019-01 onwards (end not stated) | — | — |
| AB | Allaire 2025 (55) | Western | 545 | prospective | 18.9 | 26.6 | 69 | 86.0 | 16.0 | 19.0 | 55 | 59.0 | 29.0 | 20.0 | 23.1 | 5.2 | 34.0 | — | 5 | Moderate | 545 | 545 | RECIST 1.1 | 2020-07 to 2023-01 | — | — |
| AB | Ohara 2025 (56) | Asian | 257 | retrospective | 48.2 | 47.5 | 73 | 80.2 | 52.6 | 11.7 | 116 | — | 15.2 | 33.9 | 17.5 | 5.5 | — | — | 4 | Low | 257 | 257 | mRECIST | 2020-10 to 2022-01 | 71.8 | 18.3 |
| DT | Chamseddine 2024 (57) | Western | 16 | prospective | 5 | 75 | — | 87.5 | — | 50 | — | 6.3 | — | 100 | — | 2.5 | — | — | 4 | Low | 16 | 16 | mRECIST | 2022-10 to 2024-02 | — | — |
| DT | Kawamura 2025 (58) | Asian | 21 | retrospective | 5.5 | 81 | 73 | 81 | 52 | 0 | 1053 | — | 43 | 29 | — | — | **23.8** | **47.6** | 5 | Moderate | 21 | 21 | mRECIST | 2023-05 to 2024-12 | — | — |
| DT | Fujii 2025 (59) | Asian | 21 | retrospective | 8.8 | 0 | 73 | 85.7 | 57.1 | 0 | 19.9 | — | 33.3 | 33.3 | — | 6.8 | 52.4 | 71.4 | 5 | Moderate | 21 | 21 | RECIST 1.1 + mRECIST | 2023-04 to 2024-03 | — | — |
| DT | Fujiwara 2024 (60) | Asian | 22 | retrospective | 7.6 | 54.5 | 75.6 | 86 | 59 | 10 | 36.8 | 90 | 40 | 50 | — | 3 | 9.1 | 59.1 | 4 | Low | 22 | 22 | RECIST 1.1 + mRECIST | 2023-04 to 2024-03 | 63.6 | 18.1 |
| DT | Honma 2025 (61) | Asian | 30 | retrospective | 11.1 | 50 | 75 | 80 | 53.3 | 10 | 16.7 | 86.7 | 26.7 | 43.3 | — | 3.7 | 30 | 53.3 | 5 | Moderate | 30 | 30 | RECIST 1.1 | 2023-03 to 2024-10 | 73.3 | 36.6 |
| DT | Uchikawa 2024 (62) | Asian | 33 | retrospective | — | 57.6 | 72 | 87.9 | 45.5 | 0 | 34.5 | — | 18.2 | 51.5 | — | — | 30.3 | 66.7 | 4 | Low | 33 | 33 | RECIST 1.1 + mRECIST | Not reported | — | — |
| DT | Kuzuya 2024 (63) | Asian | 40 | retrospective | 7.6 | 55 | 75 | 82.5 | 37.5 | 32.5 | 108 | 82.5 | 25 | 32.5 | — | 5.7 | 25 | 57.5 | 5 | Moderate | 40 | 40 | RECIST 1.1 + mRECIST | 2023-03 to 2024-11 | — | 25.0 |
| DT | Suzuki 2025 (64) | Asian | 64 | retrospective | 7.2 | 52 | 74 | 88 | 34 | 13 | 43.3 | 94 | — | 44 | — | 3.9 | 11 | 53 | 4 | Low | 64 | 64 | RECIST 1.1 | to 2024-12 (start not stated) | — | — |
| DT | **Sakane 2026 (65)** | Asian | 70 | retrospective | 8.8 | 65.7 | 74 | 87.1 | 32.9 | 15.7 | 52.8 | 71.4 | 25.7 | 40 | 16.2 | 2.8 | 25.8 | 59.7 | 4 | Low | 70 | **62** | RECIST 1.1 | 2023-03 to 2024-07 | — | 28.6 |
| DT | Tomonari 2025 (66) | Asian | 212 | retrospective | 6.7 | 55.2 | 74 | **80.7** | 53.7 | 10.85 | 55 | 83 | 20.8 | 38.2 | — | 3.7 | 18.9 | 51.9 | 4 | Low | 212 | 212 | RECIST 1.1 + mRECIST | 2023-03 to 2024-11 | — | — |
| DT | Celsa 2025 (67) | Western | 233 | retrospective | 6 | 20 | 69.4 | 84 | 36.4 | 30.1 | — | 49.8 | 30.9 | 30.4 | 20.4 | 6 | 21.4 | 59.4 | 4 | Low | 233 | 233 | RECIST 1.1 | 2022-01 to 2025-02 | 76.8 | 16.3 |
| DT | Matono 2025 (68) | Asian | 345 | retrospective | 7.9 | 62 | 73 | 83.8 | 45.5 | **21.4** | 55.6 | **78.0** | — | — | 17.9 | 3.9 | 19 | 49.3 | 5 | Moderate | **240** | **211** | Not reported | 2023-02 to 2024-12 | — | — |
| DT | Mohri 2026 (69) | Asian | 19 | prospective | — | **63.2** | 69 | 57.9 | 47.4 | 36.8 | — | — | 10.5 | — | — | — | 15.8 | 63.2 | 4 | Low | 19 | 19 | mRECIST | 2023-06 to 2025-04 | — | — |
| **DT** | **Bansal 2026 (70)** | **Western** | **107** | **retrospective** | **9.4** | **0** | **72.2** | **100** | **59.8** | **24.3** | **110.2** | **—** | **37.4** | **22.4** | **9.6** | **—** | **—** | **—** | **4** | **Low** | 107 | 107 | Not reported | 2008-01 to 2024-02 (HCC diagnosis window; STRIDE dates not stated) | — | — |
| **DT** | **Korphaisarn 2026 (71)** | **Asian** | **50** | **retrospective** | **28.9** | **0** | **62** | **80** | **74** | **8** | **—** | **40** | **24** | **—** | **10.5** | **4.6** | **12** | **60** | **4** | **Low** | 50 | **48** | RECIST 1.1 | 2023-08 to 2025-11 | 76.0 | 24.0 |
| **DT** | **Mıdık 2026 (72)** | **Western** | **32** | **retrospective** | **—** | **0** | **64** | **68.8** | **46.9** | **3.1** | **—** | **40.6** | **25** | **50** | **11.4** | **7.3** | **34.4** | **50** | **4** | **Low** | 32 | 32 | RECIST 1.1 | 2020-01 to 2024-12 | 34.4 | 9.4 |
| **AB** | **Haghnejad 2023 (73)** | **Western** | **50** | **retrospective** | **18.2** | **100** | **65** | **76** | **46** | **14** | **—** | **56** | **40** | **62** | **17.1** | **8** | **14** | **56** | **4** | **Low** | 50 | 50 | RECIST 1.1 | 2020-04 to 2021-11 | — | — |
| **AB** | **Dung 2025 (74)** | **Asian** | **97** | **prospective** | **11.1** | **—** | **57** | **90.7** | **100** | **3.1** | **—** | **57.7** | **48.5** | **37.1** | **20** | **4.3** | **24.7** | **64.9** | **4** | **Low** | 97 | 97 | RECIST 1.1 | 2020-06 to 2024-08 | — | — |
| **AB** | **Phillips 2026 (75)** | **Western** | **233** | **retrospective** | **17.4** | **0** | **68** | **86.3** | **17.6** | **11.1** | **—** | **26.6** | **20.2** | **25.8** | **16.5** | **12.3** | **42.9** | **—** | **4** | **Low** | 233 | 233 | RECIST 1.1 | to 2025-08 (start not stated) | 41.2 | 23.2 |
| **AB** | **Tovoli 2026 (76)** | **Western** | **538** | **prospective** | **24.2** | **0** | **69.8** | **81.4** | **60** | **10** | **—** | **69.3** | **36.2** | **—** | **19.7** | **9.7** | **29.8** | **71.2** | **5** | **Moderate** | 538 | **502** | RECIST 1.1 | to 2025-07 (start not stated) | 89.6 | 36.8 |

mOS, median overall survival; mPFS, median progression-free survival; ORR, objective response rate; DCR, disease control rate; MVI, macrovascular invasion; EHS, extrahepatic spread; ECOG, Eastern Cooperative Oncology Group; AFP, alpha-fetoprotein; NOS, Newcastle–Ottawa Scale; RECIST, Response Evaluation Criteria in Solid Tumors; mRECIST, modified RECIST; AB, atezolizumab–bevacizumab; DT, durvalumab–tremelimumab.

**Table S3. Sensitivity analyses of the adjusted treatment coefficient**

| **Analysis** | **mPFS, months (AB − DT)** | **DCR, points (AB − DT)** |
| --- | --- | --- |
| **Analysis set** | | |
| (a) Expanded 79-report set using submitted extraction values and analysis method | +2.17 (+1.21 to +3.13); P<0.001; k=55 | +15.90 (+10.50 to +21.30); P<0.001; k=52 |
| (b) Data errors corrected — 79 reports | +2.17 (+1.21 to +3.13); P<0.001; k=55 | +16.02 (+10.60 to +21.44); P<0.001; k=52 |
| (c) Inoue 2025 removed as a duplicate — 78 reports | +2.06 (+1.05 to +3.06); P<0.001; k=54 | +15.97 (+10.37 to +21.58); P<0.001; k=51 |
| (d) Interventional trials also removed — 76 reports (primary) | +1.96 (+0.91 to +3.02); P<0.001; k=52 | +16.15 (+10.44 to +21.86); P<0.001; k=49 |
| (e) Primary minus Fujii 2025 | +2.20 (+1.09 to +3.31); P<0.001; k=51 | +17.05 (+11.16 to +22.93); P<0.001; k=48 |
| (f) Primary minus Sakane 2026 | +1.79 (+0.67 to +2.91); P=0.002; k=51 | +16.29 (+10.35 to +22.23); P<0.001; k=48 |
| (g) Primary minus Tomonari 2025 | +1.84 (+0.71 to +2.98); P=0.001; k=51 | +14.57 (+8.54 to +20.60); P<0.001; k=48 |
| (h) Primary minus Honma 2025 | +1.89 (+0.78 to +3.00); P<0.001; k=51 | +15.76 (+9.90 to +21.62); P<0.001; k=48 |
| (i) Primary minus all four simultaneously | +1.69 (+0.31 to +3.07); P=0.016; k=48 | +14.78 (+7.86 to +21.71); P<0.001; k=45 |
| (j) One report retained per authorship-overlap family — 53 of 76 reports | +2.37 (+0.92 to +3.82); P=0.001; k=35 | +15.07 (+8.09 to +22.05); P<0.001; k=32 |
| **Leave-one-DT-report-out** | | |
| Leave-one-DT-report-out (mPFS) | +1.67 to +2.20; max P=0.005 |  |
| Leave-one-DT-report-out (DCR) |  | +14.57 to +17.05; max P<0.001 |
| **Treatment-line gradient (primary set)** | | |
| later-line ≤ 40% | +1.37 (−0.20 to +2.93); P=0.088; k=38 (DT 4) | +12.59 (+3.36 to +21.82); P=0.008; k=31 (DT 4) |
| later-line ≤ 30% (primary definition) | +1.35 (−0.33 to +3.04); P=0.116; k=30 (DT 4) | +14.70 (+5.12 to +24.29); P=0.003; k=22 (DT 4) |
| later-line ≤ 26% | +1.41 (−0.24 to +3.07); P=0.094; k=28 (DT 4) | +16.84 (+6.75 to +26.93); P=0.001; k=21 (DT 4) |
| later-line ≤ 20% | +1.42 (−0.31 to +3.16); P=0.107; k=27 (DT 4) | +17.89 (+6.75 to +29.03); P=0.002; k=20 (DT 4) |
| later-line ≤ 10% | +1.48 (−0.45 to +3.40); P=0.132; k=24 (DT 3) | +12.90 (+0.36 to +25.45); P=0.044; k=18 (DT 3) |
| **Source-discrepancy sensitivity** | | |
| Primary (DCR 59.7% as reported by the authors, 37/62) | — | +16.15 (+10.44 to +21.86); P<0.001; k=49 |
| Sensitivity (DCR 58.1% from CR+PR+SD, 36/62) | — | +16.30 (+10.59 to +22.01); P<0.001; k=49 |
| **Response-criteria sensitivity** | | |
| All 11 reports using mRECIST alone removed; Matono 2025, whose criterion is not stated, does not contribute to the response models | +1.98 (+0.93 to +3.02); P<0.001; k=45 | +16.06 (+10.15 to +21.97); P<0.001; k=47 (AB/DT 37 / 10) |
| **Weighting-scheme sensitivity** | | |
| Random-effects meta-regression, median/(2√n) (primary) | +1.96 (+0.91 to +3.02); P<0.001 | — |
| Random-effects meta-regression, median/√n | +1.97 (+0.86 to +3.09); P<0.001 | — |
| Random-effects meta-regression, binomial variance (primary) | — | +16.15 (+10.44 to +21.86); P<0.001 |
| Random-effects meta-regression, 1/√n | +1.98 (+0.70 to +3.26); P=0.002 | +16.84 (+8.97 to +24.71); P<0.001 |
| Sample-size-weighted least squares, HC3 robust SE | +1.79 (+0.37 to +3.22); P=0.014 | +16.80 (+9.87 to +23.73); P<0.001 |
| Unweighted least squares, HC3 robust SE | +1.98 (+0.71 to +3.24); P=0.002 | +14.65 (+9.08 to +20.21); P<0.001 |

Model and estimator as in Table 2. The 79-report rows represent the 72-report submitted set plus seven reports identified by the corrected search, before removal of one duplicate and two interventional trials. AB, atezolizumab–bevacizumab; DT, durvalumab–tremelimumab; CI, confidence interval. The weighting-scheme block reports the treatment coefficient under alternative within-report variance specifications; the two least-squares specifications use no survival variance approximation.

**Table S4. Univariate meta-regression, combined cohort**

| **Outcome** | **Covariate** | **No. of Reports** | **Coefficient (95% CI)** | **P-value** |
| --- | --- | --- | --- | --- |
| mOS | Regimen (AB vs DT) | 45 | 2.252 (−1.489, 5.994) | 0.238 |
|  | Follow-up (mo) | 38 | −0.039 (−0.174, 0.097) | 0.575 |
|  | Later-line (%) | 43 | 0.064 (0.010, 0.118) | 0.019 |
|  | Age (years) | 45 | 0.283 (0.067, 0.498) | 0.010 |
|  | Male (%) | 45 | −0.015 (−0.181, 0.152) | 0.863 |
|  | Viral (%) | 44 | −0.094 (−0.162, −0.026) | 0.007 |
|  | Child-Pugh B (%) | 43 | 0.034 (−0.095, 0.164) | 0.604 |
|  | AFP (ng/mL) | 23 | −0.001 (−0.002, 0.000) | 0.063 |
|  | ECOG 0 (%) | 32 | 0.093 (0.029, 0.158) | 0.005 |
|  | MVI (%) | 38 | −0.086 (−0.153, −0.019) | 0.012 |
|  | EHS (%) | 39 | −0.024 (−0.100, 0.052) | 0.535 |
| mPFS | Regimen (AB vs DT) | 62 | 2.243 (1.301, 3.185) | <0.001 |
|  | Follow-up (mo) | 54 | 0.024 (−0.028, 0.077) | 0.364 |
|  | Later-line (%) | 60 | −0.028 (−0.044, −0.013) | <0.001 |
|  | Age (years) | 60 | 0.001 (−0.078, 0.080) | 0.980 |
|  | Male (%) | 62 | −0.053 (−0.135, 0.029) | 0.207 |
|  | Viral (%) | 59 | −0.022 (−0.046, 0.002) | 0.076 |
|  | Child-Pugh B (%) | 60 | −0.009 (−0.047, 0.029) | 0.653 |
|  | AFP (ng/mL) | 39 | −0.000 (−0.001, 0.000) | 0.106 |
|  | ECOG 0 (%) | 44 | −0.008 (−0.030, 0.014) | 0.465 |
|  | MVI (%) | 54 | −0.024 (−0.051, 0.003) | 0.078 |
|  | EHS (%) | 57 | −0.031 (−0.056, −0.006) | 0.016 |
| ORR | Regimen (AB vs DT) | 60 | 5.038 (−0.839, 10.915) | 0.093 |
|  | Follow-up (mo) | 47 | 0.131 (−0.221, 0.484) | 0.465 |
|  | Later-line (%) | 59 | −0.116 (−0.212, −0.020) | 0.017 |
|  | Age (years) | 60 | 0.187 (−0.250, 0.623) | 0.402 |
|  | Male (%) | 60 | 0.194 (−0.245, 0.633) | 0.386 |
|  | Viral (%) | 59 | −0.177 (−0.298, −0.056) | 0.004 |
|  | Child-Pugh B (%) | 59 | 0.109 (−0.100, 0.318) | 0.307 |
|  | AFP (ng/mL) | 41 | −0.001 (−0.003, 0.001) | 0.268 |
|  | ECOG 0 (%) | 42 | −0.072 (−0.186, 0.043) | 0.219 |
|  | MVI (%) | 54 | −0.145 (−0.277, −0.013) | 0.031 |
|  | EHS (%) | 54 | −0.107 (−0.268, 0.054) | 0.191 |
| DCR | Regimen (AB vs DT) | 56 | 14.966 (9.319, 20.613) | <0.001 |
|  | Follow-up (mo) | 44 | −0.007 (−0.445, 0.431) | 0.974 |
|  | Later-line (%) | 55 | −0.051 (−0.164, 0.062) | 0.374 |
|  | Age (years) | 56 | 0.642 (0.149, 1.136) | 0.011 |
|  | Male (%) | 56 | −0.077 (−0.574, 0.420) | 0.763 |
|  | Viral (%) | 55 | −0.119 (−0.295, 0.058) | 0.188 |
|  | Child-Pugh B (%) | 56 | −0.015 (−0.255, 0.224) | 0.901 |
|  | AFP (ng/mL) | 40 | −0.002 (−0.005, 0.000) | 0.084 |
|  | ECOG 0 (%) | 38 | 0.104 (−0.049, 0.257) | 0.184 |
|  | MVI (%) | 50 | −0.307 (−0.474, −0.140) | <0.001 |
|  | EHS (%) | 50 | −0.128 (−0.322, 0.067) | 0.199 |

Random-effects meta-regression fitted with one covariate at a time; DerSimonian–Laird between-report variance with normal-approximation inference. Coefficients are per unit of the covariate and are report-level (ecological) associations. mOS, median overall survival; mPFS, median progression-free survival; ORR, objective response rate; DCR, disease control rate; CI, confidence interval; MVI, macrovascular invasion; EHS, extrahepatic spread.

**Table S5. Univariate meta-regression, regimen-specific**

| **Group** | **Outcome** | **Covariate** | **No. of Reports** | **Coefficient (95% CI)** | **P-value** |
| --- | --- | --- | --- | --- | --- |
| AB | mOS | Follow-up (mo) | 33 | −0.017 (−0.159, 0.125) | 0.814 |
|  |  | Later-line (%) | 37 | 0.063 (0.002, 0.124) | 0.044 |
|  |  | Age (years) | 39 | 0.285 (0.064, 0.506) | 0.012 |
|  |  | Male (%) | 39 | −0.004 (−0.193, 0.186) | 0.971 |
|  |  | Viral (%) | 38 | −0.087 (−0.159, −0.016) | 0.017 |
|  |  | Child-Pugh B (%) | 37 | 0.013 (−0.124, 0.149) | 0.853 |
|  |  | AFP (ng/mL) | 20 | −0.001 (−0.002, −0.000) | 0.025 |
|  |  | ECOG 0 (%) | 27 | 0.089 (0.019, 0.158) | 0.012 |
|  |  | MVI (%) | 33 | −0.095 (−0.161, −0.029) | 0.005 |
|  |  | EHS (%) | 35 | −0.028 (−0.104, 0.048) | 0.473 |
|  | mPFS | Follow-up (mo) | 43 | 0.002 (−0.049, 0.053) | 0.948 |
|  |  | Later-line (%) | 48 | −0.008 (−0.027, 0.012) | 0.438 |
|  |  | Age (years) | 49 | 0.067 (−0.013, 0.147) | 0.100 |
|  |  | Male (%) | 50 | −0.019 (−0.102, 0.064) | 0.652 |
|  |  | Viral (%) | 48 | −0.031 (−0.054, −0.008) | 0.008 |
|  |  | Child-Pugh B (%) | 48 | 0.008 (−0.032, 0.049) | 0.688 |
|  |  | AFP (ng/mL) | 31 | −0.000 (−0.001, −0.000) | 0.011 |
|  |  | ECOG 0 (%) | 33 | 0.000 (−0.025, 0.026) | 0.994 |
|  |  | MVI (%) | 45 | −0.032 (−0.056, −0.007) | 0.011 |
|  |  | EHS (%) | 47 | −0.020 (−0.046, 0.006) | 0.138 |
|  | ORR | Follow-up (mo) | 36 | 0.168 (−0.231, 0.566) | 0.410 |
|  |  | Later-line (%) | 45 | −0.101 (−0.222, 0.019) | 0.098 |
|  |  | Age (years) | 46 | 0.316 (−0.157, 0.789) | 0.190 |
|  |  | Male (%) | 46 | 0.297 (−0.248, 0.841) | 0.285 |
|  |  | Viral (%) | 45 | −0.211 (−0.333, −0.089) | <0.001 |
|  |  | Child-Pugh B (%) | 45 | 0.195 (−0.036, 0.427) | 0.098 |
|  |  | AFP (ng/mL) | 31 | −0.001 (−0.004, 0.001) | 0.230 |
|  |  | ECOG 0 (%) | 32 | −0.018 (−0.140, 0.104) | 0.771 |
|  |  | MVI (%) | 42 | −0.173 (−0.307, −0.040) | 0.011 |
|  |  | EHS (%) | 43 | −0.105 (−0.272, 0.061) | 0.215 |
|  | DCR | Follow-up (mo) | 33 | −0.188 (−0.592, 0.217) | 0.363 |
|  |  | Later-line (%) | 41 | 0.071 (−0.039, 0.182) | 0.206 |
|  |  | Age (years) | 42 | 0.982 (0.582, 1.381) | <0.001 |
|  |  | Male (%) | 42 | −0.147 (−0.668, 0.374) | 0.580 |
|  |  | Viral (%) | 41 | −0.224 (−0.377, −0.071) | 0.004 |
|  |  | Child-Pugh B (%) | 42 | −0.020 (−0.241, 0.201) | 0.857 |
|  |  | AFP (ng/mL) | 30 | −0.003 (−0.005, −0.000) | 0.016 |
|  |  | ECOG 0 (%) | 28 | 0.232 (0.138, 0.327) | <0.001 |
|  |  | MVI (%) | 38 | −0.378 (−0.522, −0.234) | <0.001 |
|  |  | EHS (%) | 39 | −0.139 (−0.311, 0.033) | 0.114 |
| DT | mOS | Follow-up (mo) | 5 | −0.310 (−0.841, 0.222) | 0.254 |
|  |  | Later-line (%) | 6 | 0.095 (−0.017, 0.207) | 0.095 |
|  |  | Age (years) | 6 | 0.416 (−0.516, 1.348) | 0.381 |
|  |  | Male (%) | 6 | −0.024 (−0.479, 0.430) | 0.916 |
|  |  | Viral (%) | 6 | −0.223 (−0.404, −0.043) | 0.015 |
|  |  | Child-Pugh B (%) | 6 | 0.256 (−0.176, 0.689) | 0.245 |
|  |  | ECOG 0 (%) | 5 | 0.140 (−0.106, 0.387) | 0.265 |
|  |  | MVI (%) | 5 | −0.041 (−1.119, 1.036) | 0.940 |
|  | mPFS | Follow-up (mo) | 11 | 0.023 (−0.080, 0.126) | 0.659 |
|  |  | Later-line (%) | 12 | −0.044 (−0.061, −0.028) | <0.001 |
|  |  | Age (years) | 11 | −0.162 (−0.290, −0.033) | 0.014 |
|  |  | Male (%) | 12 | −0.176 (−0.309, −0.043) | 0.010 |
|  |  | Viral (%) | 11 | −0.001 (−0.059, 0.056) | 0.963 |
|  |  | Child-Pugh B (%) | 12 | −0.031 (−0.081, 0.019) | 0.220 |
|  |  | AFP (ng/mL) | 8 | 0.012 (−0.009, 0.032) | 0.270 |
|  |  | ECOG 0 (%) | 11 | −0.006 (−0.031, 0.019) | 0.622 |
|  |  | MVI (%) | 9 | −0.012 (−0.194, 0.170) | 0.899 |
|  |  | EHS (%) | 10 | −0.040 (−0.081, −0.000) | 0.050 |
|  | ORR | Follow-up (mo) | 11 | −0.306 (−0.963, 0.351) | 0.361 |
|  |  | Later-line (%) | 14 | −0.072 (−0.249, 0.105) | 0.424 |
|  |  | Age (years) | 14 | 0.102 (−0.960, 1.164) | 0.851 |
|  |  | Male (%) | 14 | −0.062 (−0.686, 0.562) | 0.846 |
|  |  | Viral (%) | 14 | −0.110 (−0.470, 0.250) | 0.550 |
|  |  | Child-Pugh B (%) | 14 | −0.190 (−0.588, 0.209) | 0.351 |
|  |  | AFP (ng/mL) | 10 | 0.002 (−0.021, 0.024) | 0.881 |
|  |  | ECOG 0 (%) | 10 | −0.089 (−0.298, 0.119) | 0.401 |
|  |  | MVI (%) | 12 | −0.001 (−0.650, 0.649) | 0.998 |
|  |  | EHS (%) | 11 | −0.209 (−0.943, 0.526) | 0.577 |
|  | DCR | Follow-up (mo) | 11 | 0.217 (−0.440, 0.873) | 0.518 |
|  |  | Later-line (%) | 14 | −0.126 (−0.260, 0.009) | 0.068 |
|  |  | Age (years) | 14 | −0.510 (−1.429, 0.408) | 0.276 |
|  |  | Male (%) | 14 | 0.138 (−0.487, 0.763) | 0.665 |
|  |  | Viral (%) | 14 | −0.041 (−0.355, 0.274) | 0.800 |
|  |  | Child-Pugh B (%) | 14 | 0.031 (−0.284, 0.346) | 0.847 |
|  |  | AFP (ng/mL) | 10 | −0.008 (−0.030, 0.014) | 0.489 |
|  |  | ECOG 0 (%) | 10 | −0.152 (−0.336, 0.033) | 0.107 |
|  |  | MVI (%) | 12 | 0.111 (−0.446, 0.669) | 0.696 |
|  |  | EHS (%) | 11 | −0.112 (−0.634, 0.411) | 0.675 |

As Table S4, fitted separately within each regimen arm. Abbreviations as in Table S4.

**Table S6. Real-world cohorts directly comparing AB and DT**

| **Report** | **Data source and design** | **AB / DT (analyzed)** | **Within original search window?** | **Overall survival** | **Other outcomes** |
| --- | --- | --- | --- | --- | --- |
| Lo Prinzi F, et al. Target Oncol 2025; doi:10.1007/s11523-025-01161-5; online 23 Jun 2025 | TriNetX federated network; propensity-score matched | 309 / 309 | Yes — missed | mOS 15.4 vs 15.5 months; HR 0.94 (0.73–1.22); P=0.67 | not reported |
| Shiraishi C, et al. Front Oncol 2026; doi:10.3389/fonc.2026.1776032; online 22 Jan 2026 | TriNetX Research Network; target trial emulation; treated Nov 2022–Nov 2024, censored 30 Nov 2025; propensity-score matched (1,394 / 698 before matching) | 640 / 640 | Yes — missed | mOS 19.4 vs 19.0 months; HR 0.886 (0.743–1.057); P=0.179. 1-year OS 61% vs 55%; HR 0.806 (0.665–0.976); P=0.027; 2-year 43.9% vs 42.9%; P=0.105 | Hepatic irAE HR 0.678 (0.487–0.943); P=0.020; respiratory irAE onset earlier with AB |
| Ailawadi S, et al. Biomedicines 2026;14(7):1638; doi:10.3390/biomedicines14071638; online 21 Jul 2026 | TriNetX (112 healthcare organisations); data accessed 7 May 2026; propensity-score matched (2,031 / 788 before matching) | 768 / 768 | No — post-dates window | mOS 16.3 vs 22.5 months; HR 0.89 (0.76–1.0); not significant | immune-mediated colitis 3.0% vs 5.1%; hospitalisation similar |
| Kournoutas I, et al. Oncologist 2025;30(11):oyaf286; online 18 Sep 2025 | 6-centre retrospective; first line. Comparator arm is durvalumab ± tremelimumab — durvalumab monotherapy is included | 336 / 116 | Yes — missed | mOS 14.0 vs 14.6 months; P=0.664 | ORR 26.3% vs 22.0% (P=0.375); DCR 61.5% vs 56.0% (P=0.306) |
| Ohama H, et al. Hepatol Res 2026;56:716–724; online 9 Jan 2026 | Multicenter retrospective (RELPEC / HCC 48); Child-Pugh B only; inverse-probability weighted | 211 total | Yes — missed | mOS 10.5 vs 12.4 months; not significant after weighting | mPFS 5.0 vs 3.5 months; DCR 75.2% vs 55.0% (P=0.02) |
| Hiraoka A, et al. Liver Cancer 2026; doi:10.1159/000550924; online 2 Apr 2026 | Multicenter retrospective (RELPEC / HCC 48); first line; Child-Pugh A, BCLC-B/C; enrolled 2023–2025; inverse-probability weighted | 302 / 129 | No — post-dates window | mOS 25.6 vs 22.1 months; P=0.172 | mPFS 9.1 vs 5.0 months (P=0.002); ORR 31.8% vs 28.7% (P=0.570); DCR 71.9% vs 63.6% (P=0.110) |
| Lin SH, et al. J Gastroenterol Hepatol 2026; doi:10.1111/jgh.70555; online 10 Jul 2026 | TriNetX Global Collaborative Network; first-line therapy started 1 Oct 2022–1 Oct 2025; propensity-score matched 1:1 (3,362 eligible) | 765 / 765 | No — post-dates window | mOS 14.6 vs 17.3 months; adjusted HR 0.96 (0.83–1.12); median follow-up 14.8 vs 17.5 months | Treatment-requiring irAE HR 0.61 (0.48–0.77) favoring AB; GI bleeding HR 0.88 (0.68–1.13); non-GI bleeding HR 1.06 (0.60–1.89) |
| Tanaka K, et al. Liver Int 2026;46:e70803; doi:10.1111/liv.70803; online 1 Aug 2026 | Multicenter retrospective, Japan (RELPEC / HCC 48 investigators); first line 2020–2025; training and validation cohorts; spline interaction models | 1,464 total | No — post-dates window | not reported | Overall ORR did not differ between regimens. Baseline des-γ-carboxy prothrombin modified the ORR difference: AB favored at low levels, DT increasingly favored as levels rise |
| Ben Khaled N, et al. JHEP Rep 2026;8:101818; doi:10.1016/j.jhepr.2026.101818; online 11 Mar 2026 | 11 tertiary centres, Europe; retrospective; AB treated before DT approval; safety endpoints only | 325 / 165 | No — post-dates window | not reported | Bleeding of any grade 22.8% (AB) vs 13.3% (DT), P=0.016; grade ≥3 bleeding 14.5% vs 7.3%, P=0.027; shorter time to first bleeding with AB (P=0.037), independent on multivariable analysis (P=0.010). Variceal bleeding and thromboembolic events did not differ |

These reports compare the two regimens within a single source population and are presented separately rather than pooled with the primary single-arm evidence. Serial reports from shared federated or multicenter networks mean that the nine reports represent four independent source families. Two were retrieved by the corrected primary database search and are included among the full-text exclusions in Figure S1; seven additional comparator-focused records were identified only by the targeted search and lie outside the primary screening flow. None was pooled with or counted among the 76 primary reports. AB, atezolizumab–bevacizumab; DT, durvalumab–tremelimumab; mOS, median overall survival; mPFS, median progression-free survival; ORR, objective response rate; DCR, disease control rate; HR, hazard ratio; irAE, immune-related adverse event.

**Table S7. Exploratory model performance (pooled out-of-fold cross-validation)**

| **Outcome** | **Model** | **Out-of-fold R²** | **SD** | **RMSE** | **MAE** | **RMSE vs baseline** |
| --- | --- | --- | --- | --- | --- | --- |
| mPFS | Ridge | +0.147 | 0.072 | 1.79 | 1.38 | −9.6% |
| mPFS | Elastic net | +0.114 | 0.066 | 1.83 | 1.40 | −7.8% |
| mPFS | SVR | +0.078 | 0.055 | 1.87 | 1.39 | −5.9% |
| mPFS | LASSO | +0.054 | 0.061 | 1.89 | 1.44 | −4.7% |
| mPFS | Random forest | +0.001 | 0.085 | 1.94 | 1.50 | −2.1% |
| mPFS | Intercept-only baseline | −0.041 | 0.023 | 1.98 | 1.47 | +0.0% |
| mPFS | Gradient boosting | −0.199 | 0.071 | 2.13 | 1.60 | +7.3% |
| DCR | SVR | +0.376 | 0.016 | 9.57 | 6.61 | −22.3% |
| DCR | Random forest | +0.266 | 0.067 | 10.37 | 7.43 | −15.8% |
| DCR | Ridge | +0.260 | 0.031 | 10.43 | 7.69 | −15.4% |
| DCR | Gradient boosting | +0.218 | 0.065 | 10.71 | 7.77 | −13.1% |
| DCR | Elastic net | +0.212 | 0.053 | 10.75 | 7.91 | −12.8% |
| DCR | LASSO | +0.194 | 0.036 | 10.88 | 7.68 | −11.7% |
| DCR | Intercept-only baseline | −0.034 | 0.014 | 12.33 | 9.63 | +0.0% |

Pooled out-of-fold R² from repeated stratified five-fold cross-validation (six repeats) with hyperparameters tuned in an inner four-fold loop and all preprocessing performed inside each fold. AB, atezolizumab–bevacizumab; DT, durvalumab–tremelimumab; RMSE, root mean squared error; MAE, mean absolute error; SVR, support-vector regression; SD, standard deviation.

**Table S8. Exploratory scenario-level model-predicted mPFS differences (T-learner, supporting analysis)**

| **Scenario** | **AB (months)** | **DT (months)** | **Difference, months (bootstrap variability interval)** | **DT covariate support** |
| --- | --- | --- | --- | --- |
| HIMALAYA-like | 8.9 | 5.7 | +3.2 (+1.1 to +6.2) | extrapolated: viral, MVI, EHS |
| IMbrave150-like | 7.2 | 5.7 | +1.5 (+0.2 to +4.5) | extrapolated: EHS |
| Advanced | 6.0 | 5.3 | +0.7 (−0.8 to +3.8) | extrapolated: MVI, EHS |
| Non-advanced | 7.4 | 5.4 | +2.0 (+0.6 to +3.9) | extrapolated: viral, MVI, EHS |
| First-line | 7.2 | 5.7 | +1.5 (+0.4 to +3.8) | within support |
| Later-line | 6.4 | 3.5 | +3.0 (−0.3 to +4.2) | extrapolated: later-line |
| Asian RW | 6.3 | 5.2 | +1.1 (+0.1 to +2.7) | extrapolated: viral |
| Western RW | 7.8 | 5.3 | +2.5 (+0.8 to +4.4) | within support |
| CP-A dominant | 7.0 | 5.3 | +1.7 (+0.5 to +3.5) | within support |
| CP-B included | 6.8 | 5.2 | +1.5 (+0.4 to +3.2) | within support |

Values are model-predicted mPFS from regimen-specific models evaluated at each covariate profile, not estimated treatment effects. Bootstrap variability intervals were based on 500 resamples drawn independently within each arm. AB, atezolizumab–bevacizumab; DT, durvalumab–tremelimumab.

**Table S9. Exploratory scenario-level model-predicted DCR differences (T-learner, supporting analysis)**

| **Scenario** | **AB (%)** | **DT (%)** | **Difference, percentage points (bootstrap variability interval)** | **DT covariate support** |
| --- | --- | --- | --- | --- |
| HIMALAYA-like | 64.9 | 57.8 | +7.1 (−1.1 to +19.2) | extrapolated: viral, MVI, EHS |
| IMbrave150-like | 64.9 | 55.7 | +9.2 (−0.1 to +19.7) | extrapolated: EHS |
| Advanced | 64.2 | 61.3 | +3.0 (−4.5 to +15.1) | extrapolated: MVI, EHS |
| Non-advanced | 72.7 | 53.1 | +19.6 (+7.1 to +25.9) | extrapolated: viral, MVI, EHS |
| First-line | 70.8 | 55.6 | +15.2 (+6.1 to +22.1) | within support |
| Later-line | 70.2 | 53.3 | +16.9 (+5.3 to +22.0) | extrapolated: later-line |
| Asian RW | 75.2 | 55.4 | +19.8 (+10.6 to +24.9) | extrapolated: viral |
| Western RW | 66.1 | 53.8 | +12.3 (+5.1 to +23.4) | within support |
| CP-A dominant | 73.6 | 55.5 | +18.1 (+7.1 to +23.3) | within support |
| CP-B included | 72.4 | 53.4 | +19.0 (+10.0 to +23.4) | within support |

Values are model-predicted DCR from regimen-specific models evaluated at each covariate profile, not estimated treatment effects. Bootstrap variability intervals were based on 500 resamples drawn independently within each arm. AB, atezolizumab–bevacizumab; DT, durvalumab–tremelimumab.

**Table S10. Covariate profiles for the exploratory prediction analysis**

| **Scenario** | **Later-line (%)** | **Age (years)** | **Male (%)** | **Viral (%)** | **CP-B (%)** | **MVI (%)** | **EHS (%)** | **DT support (mPFS set)** | **DT support (DCR set)** |
| --- | --- | --- | --- | --- | --- | --- | --- | --- | --- |
| HIMALAYA-like | 0 | 65 | 83 | 30 | 0 | 10 | 55 | extrapolated: viral, MVI, EHS | extrapolated: viral, MVI, EHS |
| IMbrave150-like | 0 | 65 | 82 | 50 | 0 | 38 | 63 | extrapolated: EHS | extrapolated: EHS |
| Advanced | 20 | 65 | 85 | 55 | 5 | 60 | 70 | extrapolated: MVI, EHS | extrapolated: MVI, EHS |
| Non-advanced | 15 | 68 | 80 | 60 | 5 | 15 | 25 | extrapolated: viral, MVI, EHS | extrapolated: viral, MVI, EHS |
| First-line | 0 | 66 | 82 | 55 | 5 | 30 | 45 | within support | within support |
| Later-line | 100 | 66 | 80 | 55 | 10 | 35 | 50 | extrapolated: later-line | extrapolated: later-line |
| Asian RW | 25 | 72 | 82 | 65 | 12 | 28 | 38 | extrapolated: viral | extrapolated: viral |
| Western RW | 18 | 65 | 78 | 35 | 8 | 32 | 42 | within support | within support |
| CP-A dominant | 20 | 68 | 82 | 58 | 0 | 28 | 40 | within support | within support |
| CP-B included | 22 | 68 | 80 | 55 | 25 | 30 | 42 | within support | within support |

Profiles were specified before the exploratory analysis was run. The final two columns state, for each profile, whether every covariate value lies within the range observed in the DT arm of that analysis set. Four of the ten profiles do; six require extrapolation. EHS was the most frequent extrapolation reason (four profiles); MVI and viral status each contributed to three profiles. Because these are marginal ranges assessed one covariate at a time, they overstate joint multivariable support, and scenario estimates are illustrative of model behavior rather than inference for any clinical profile. AB, atezolizumab–bevacizumab; DT, durvalumab–tremelimumab; MVI, macrovascular invasion; EHS, extrahepatic spread.

**Reference list for the 76 primary reports and three audit/sensitivity-only reports. Entries 77–79 are Inoue 2025 (duplicate) and the interventional reports by Piscaglia 2025 and Shao 2025; they are retained only to document the eligibility audit and sensitivity analyses.**

| **No.** | **Report label** | **No. in the submitted supplement** | **Reference** |
| --- | --- | --- | --- |
| 1 | Dhampalwar 2023[1] | 1 | Dhampalwar S, Choudhary NS, Saraf N, Bhangui P, Soin AS. Atezolizumab and bevacizumab for treatment of patients with unresectable/non-transplantable advanced hepatocellular carcinoma: a real-world single center experience from north India. Journal of Clinical and Experimental Hepatology. 2023;14(1):101219. doi: 10.1016/j.jceh.2023.07.006. PubMed PMID: 38076355. |
| 2 | Yiu 2023[2] | 2 | Yiu DC, Chan BL, Wong AC, Feng MY, Chan SL. Real‐world experiences of atezolizumab plus bevacizumab in patients with advanced hepatocellular carcinoma in Hong Kong. Liver Cancer International. 2023;4(3-4):121–6. doi: 10.1002/lci2.76. |
| 3 | Nakabori 2024[3] | 3 | Nakabori T, Higashi S, Abe Y, Mukai K, Ikawa T, Konishi K, et al. Safety and Feasibility of Combining On-Demand Selective Locoregional Treatment with First-Line Atezolizumab Plus Bevacizumab for Patients with Unresectable Hepatocellular Carcinoma. Curr Oncol. 2024;31(3):1543–55. Epub 20240315. doi: 10.3390/curroncol31030117. PubMed PMID: 38534950; PubMed Central PMCID: PMCPMC10969074. |
| 4 | Kimura 2024[4] | 4 | Kimura M, Nishikawa K, Imamura J, Kimura K. Frontline evaluation: Atezolizumab-bevacizumab versus lenvatinib for BCLC stage B hepatocellular carcinoma exceeding the up-to-seven criteria. Cancer Med. 2024;13(18):e70217. doi: 10.1002/cam4.70217. PubMed PMID: 39300938; PubMed Central PMCID: PMCPMC11413408. |
| 5 | Ballester 2024[5] | 5 | Ballester MP, Abril C, Merino V, Alos M, Segarra G, Tosca J, et al. Atezolizumab Plus Bevacizumab Treatment for Unresectable Hepatocellular Carcinoma: Real-life Experience from a Single Tertiary Centre in Spain and ALBI Score as a Survival Prognostic Factor. Cancer Diagn Progn. 2024;4(5):623–30. Epub 20240901. doi: 10.21873/cdp.10373. PubMed PMID: 39238615; PubMed Central PMCID: PMCPMC11372697. |
| 6 | Tanaka 2022[6] | 6 | Tanaka T, Takata K, Yokoyama K, Fukuda H, Yamauchi R, Fukunaga A, et al. Pretreatment Modified Albumin-Bilirubin Grade Is an Important Predictive Factor Associated with the Therapeutic Response and the Continuation of Atezolizumab plus Bevacizumab Combination Therapy for Patients with Unresectable Hepatocellular Carcinoma. Curr Oncol. 2022;29(7):4799–810. Epub 20220708. doi: 10.3390/curroncol29070381. PubMed PMID: 35877241; PubMed Central PMCID: PMCPMC9324802. |
| 7 | Charonpongsuntorn 2022[7] | 7 | Charonpongsuntorn C, Tanasanvimon S, Korphaisarn K, Payapwattanawong S, Siripoon T, Pakvisal N, et al. Efficacy, Safety, and Patient-Reported Outcomes of Atezolizumab Plus Bevacizumab for Unresectable Hepatocellular Carcinoma in Thailand: A Multicenter Prospective Study. JCO Glob Oncol. 2022;8:e2200205. doi: 10.1200/GO.22.00205. PubMed PMID: 36455172; PubMed Central PMCID: PMCPMC10166432. |
| 8 | Park 2024[8] | 8 | Park J, Lee YB, Ko Y, Park Y, Shin H, Hur MH, et al. Comparison of atezolizumab plus bevacizumab and lenvatinib for hepatocellular carcinoma with portal vein tumor thrombosis. J Liver Cancer. 2024;24(1):81–91. Epub 20240119. doi: 10.17998/jlc.2023.12.25. PubMed PMID: 38246747; PubMed Central PMCID: PMCPMC10990665. |
| 9 | Matsumoto 2022[9] | 9 | Matsumoto H, Tsuchiya K, Nakanishi H, Hayakawa Y, Yasui Y, Uchihara N, et al. Clinical Usefulness of Monitoring Muscle Volume during Atezolizumab Plus Bevacizumab Therapy in Patients with Unresectable Hepatocellular Carcinoma. Cancers (Basel). 2022;14(14). Epub 20220721. doi: 10.3390/cancers14143551. PubMed PMID: 35884610; PubMed Central PMCID: PMCPMC9322937. |
| 10 | Lee 2023[10] | 10 | Lee YC, Huang WT, Lee MY, Tsao CJ, Feng YH. Bevacizumab and Atezolizumab for Unresectable Hepatocellular Carcinoma: Real-world Data in Taiwan-Tainan Medical Oncology Group H01 Trial. In Vivo. 2023;37(1):454–60. doi: 10.21873/invivo.13099. PubMed PMID: 36593061; PubMed Central PMCID: PMCPMC9843751. |
| 11 | Takada 2022[11] | 11 | Takada H, Yamashita K, Osawa L, Komiyama Y, Nakakuki N, Muraoka M, et al. Prediction of Therapeutic Response Using Contrast-Enhanced Ultrasound in Japanese Patients Treated with Atezolizumab and Bevacizumab for Unresectable Hepatocellular Carcinoma. Oncology. 2023;101(3):173–84. Epub 20221214. doi: 10.1159/000527752. PubMed PMID: 36516803. |
| 12 | Kimura 2024[12] | 12 | Kimura M, Yamada S, Go M, Yasuda S, Toyoda H, Usami E. Evaluation of Atezolizumab Plus Bevacizumab Versus Modified Lenvatinib Therapy in Child-Pugh A Unresectable Hepatocellular Carcinoma. Cancer Diagn Progn. 2024;4(2):122–8. Epub 20240303. doi: 10.21873/cdp.10297. PubMed PMID: 38434917; PubMed Central PMCID: PMCPMC10905284. |
| 13 | Mohamed 2022[13] | 13 | Mohamed YI, Duda DG, Awiwi MO, Lee SS, Altameemi L, Xiao L, et al. Plasma growth hormone is a potential biomarker of response to atezolizumab and bevacizumab in advanced hepatocellular carcinoma patients. Oncotarget. 2022;13:1314–21. Epub 20221206. doi: 10.18632/oncotarget.28322. PubMed PMID: 36473155; PubMed Central PMCID: PMCPMC9726202. |
| 14 | Ando 2021[14] | 14 | Ando Y, Kawaoka T, Kosaka M, Shirane Y, Johira Y, Miura R, et al. Early Tumor Response and Safety of Atezolizumab Plus Bevacizumab for Patients with Unresectable Hepatocellular Carcinoma in Real-World Practice. Cancers (Basel). 2021;13(16). Epub 20210805. doi: 10.3390/cancers13163958. PubMed PMID: 34439111; PubMed Central PMCID: PMCPMC8394131. |
| 15 | Eso 2021[15] | 15 | Eso Y, Takeda H, Taura K, Takai A, Takahashi K, Seno H. Pretreatment Neutrophil-to-Lymphocyte Ratio as a Predictive Marker of Response to Atezolizumab Plus Bevacizumab for Hepatocellular Carcinoma. Curr Oncol. 2021;28(5):4157–66. Epub 20211014. doi: 10.3390/curroncol28050352. PubMed PMID: 34677270; PubMed Central PMCID: PMCPMC8534657. |
| 16 | Su 2022[16] | 16 | Su CW, Teng W, Lin PT, Jeng WJ, Chen KA, Hsieh YC, et al. Similar efficacy and safety between lenvatinib versus atezolizumab plus bevacizumab as the first-line treatment for unresectable hepatocellular carcinoma. Cancer Med. 2023;12(6):7077–89. Epub 20221205. doi: 10.1002/cam4.5506. PubMed PMID: 36468578; PubMed Central PMCID: PMCPMC10067067. |
| 17 | Wang 2022[17] | 17 | Wang JH, Chen YY, Kee KM, Wang CC, Tsai MC, Kuo YH, et al. The Prognostic Value of Neutrophil-to-Lymphocyte Ratio and Platelet-to-Lymphocyte Ratio in Patients with Hepatocellular Carcinoma Receiving Atezolizumab Plus Bevacizumab. Cancers (Basel). 2022;14(2). Epub 20220111. doi: 10.3390/cancers14020343. PubMed PMID: 35053508; PubMed Central PMCID: PMCPMC8774110. |
| 18 | Toshida 2023[18] | 18 | Toshida K, Itoh S, Yoshiya S, Nagao Y, Tomino T, Izumi T, et al. Pretreatment eosinophil count predicts response to atezolizumab plus bevacizumab therapy in patients with hepatocellular carcinoma. J Gastroenterol Hepatol. 2024;39(3):576–86. Epub 20231212. doi: 10.1111/jgh.16441. PubMed PMID: 38084637. |
| 19 | Sinner 2022[19] | 19 | Sinner F, Pinter M, Scheiner B, Ettrich TJ, Sturm N, Gonzalez-Carmona MA, et al. Atezolizumab Plus Bevacizumab in Patients with Advanced and Progressing Hepatocellular Carcinoma: Retrospective Multicenter Experience. Cancers (Basel). 2022;14(23). Epub 20221202. doi: 10.3390/cancers14235966. PubMed PMID: 36497447; PubMed Central PMCID: PMCPMC9737362. |
| 20 | Hayakawa 2021[20] | 20 | Hayakawa Y, Tsuchiya K, Kurosaki M, Yasui Y, Kaneko S, Tanaka Y, et al. Early experience of atezolizumab plus bevacizumab therapy in Japanese patients with unresectable hepatocellular carcinoma in real-world practice. Invest New Drugs. 2022;40(2):392–402. Epub 20210929. doi: 10.1007/s10637-021-01185-4. PubMed PMID: 34586531. |
| 21 | Shigefuku 2024[21] | 21 | Shigefuku R, Iwasa M, Tanaka H, Tsukimoto M, Tamai Y, Fujiwara N, et al. Prognostic Significance of Psoas Muscle Index in Unresectable Hepatocellular Carcinoma: Comparative Analysis of Lenvatinib and Atezolizumab Plus Bevacizumab. J Clin Med. 2024;13(19). Epub 20241004. doi: 10.3390/jcm13195925. PubMed PMID: 39407985; PubMed Central PMCID: PMCPMC11477730. |
| 22 | Sasaki 2024[22] | 22 | Sasaki Y, Matsumoto K, Takaki A, Adachi T, Takahara M, Ozato K, et al. Anti-PD-1 Autoantibody Predicts Survival of Patients With Hepatocellular Carcinoma Receiving Atezolizumab/Bevacizumab. Gastro Hep Adv. 2024;3(8):1138–47. Epub 20240802. doi: 10.1016/j.gastha.2024.07.018. PubMed PMID: 39559295; PubMed Central PMCID: PMCPMC11570718. |
| 23 | Akyildiz 2023[23] | 23 | Akyildiz A, Guven DC, Ozluk AA, Ismayilov R, Mutlu E, Unal OU, et al. The safety and efficacy of first-line atezolizumab plus bevacizumab in patients with unresectable hepatocellular carcinoma: A multicenter real-world study from Turkey. Medicine (Baltimore). 2023;102(45):e35950. doi: 10.1097/MD.0000000000035950. PubMed PMID: 37960746; PubMed Central PMCID: PMCPMC10637501. |
| 24 | Himmelsbach 2022[24] | 24 | Himmelsbach V, Pinter M, Scheiner B, Venerito M, Sinner F, Zimpel C, et al. Efficacy and Safety of Atezolizumab and Bevacizumab in the Real-World Treatment of Advanced Hepatocellular Carcinoma: Experience from Four Tertiary Centers. Cancers (Basel). 2022;14(7). Epub 20220328. doi: 10.3390/cancers14071722. PubMed PMID: 35406493; PubMed Central PMCID: PMCPMC8996828. |
| 25 | Kuo 2025[25] | 25 | Kuo YH, Chen YH, Tsai MC, Lu SN, Hu TH, Hung CH, et al. Comparing health insurance-reimbursed lenvatinib and self-paid atezolizumab plus bevacizumab in patients with unresectable hepatocellular carcinoma. Am J Cancer Res. 2025;15(2):811–23. Epub 20250215. doi: 10.62347/BYIE2654. PubMed PMID: 40084378; PubMed Central PMCID: PMCPMC11897613. |
| 26 | Hsu 2024[26] | 26 | Hsu YC, Lin PT, Teng W, Hsieh YC, Chen WT, Su CW, et al. Comparing Lenvatinib/Pembrolizumab with Atezolizumab/Bevacizumab in Unresectable Hepatocellular Carcinoma: A Real-World Experience with Propensity Score Matching Analysis. Cancers (Basel). 2024;16(20). Epub 20241012. doi: 10.3390/cancers16203458. PubMed PMID: 39456553; PubMed Central PMCID: PMCPMC11506658. |
| 27 | Teng 2022[27] | 27 | Teng W, Lin CC, Su CW, Lin PT, Hsieh YC, Chen WT, et al. Combination of CRAFITY score with Alpha-fetoprotein response predicts a favorable outcome of atezolizumab plus bevacizumab for unresectable hepatocellular carcinoma. Am J Cancer Res. 2022;12(4):1899–911. Epub 20220415. PubMed PMID: 35530282; PubMed Central PMCID: PMCPMC9077079. |
| 28 | Hu 2024[28] | 28 | Hu Z, Yang Z, Fu Z, Fu Y, Ma Z, Zhou Z, et al. Efficacy and safety of atezolizumab-bevacizumab vs pembrolizumab-lenvatinib in unresectable hepatocellular carcinoma: a retrospective, cohort study. Front Immunol. 2024;15:1472870. Epub 20241119. doi: 10.3389/fimmu.2024.1472870. PubMed PMID: 39628474; PubMed Central PMCID: PMCPMC11611749. |
| 29 | Chuma 2021[29] | 29 | Chuma M, Uojima H, Hattori N, Arase Y, Fukushima T, Hirose S, et al. Safety and efficacy of atezolizumab plus bevacizumab in patients with unresectable hepatocellular carcinoma in early clinical practice: A multicenter analysis. Hepatol Res. 2022;52(3):269–80. Epub 20211130. doi: 10.1111/hepr.13732. PubMed PMID: 34761470. |
| 30 | Suzuki 2024[30] | 30 | Suzuki T, Matsuura K, Suzuki Y, Okumura F, Nagura Y, Sobue S, et al. Serum interleukin-6 levels at the start of the second course of atezolizumab plus bevacizumab therapy predict therapeutic efficacy in patients with advanced hepatocellular carcinoma: a multicenter analysis. J Gastroenterol Hepatol. 2024;39(10):2158–68. Epub 20240628. doi: 10.1111/jgh.16672. PubMed PMID: 38943340. |
| 31 | Jost-Brinkmann 2023[31] | 31 | Jost-Brinkmann F, Demir M, Wree A, Luedde T, Loosen SH, Muller T, et al. Atezolizumab plus bevacizumab in unresectable hepatocellular carcinoma: Results from a German real-world cohort. Aliment Pharmacol Ther. 2023;57(11):1313–25. Epub 20230308. doi: 10.1111/apt.17441. PubMed PMID: 36883351. |
| 32 | Kinami 2023[32] | 32 | Kinami T, Amioka K, Kawaoka T, Uchikawa S, Yamasaki S, Kosaka M, et al. Evaluation of Response to Atezolizumab Plus Bevacizumab in Patients with Advanced Hepatocellular Carcinoma Using the Combination of Response Evaluation Criteria in Solid Tumors and Alpha-Fetoprotein. Cancers (Basel). 2023;15(8). Epub 20230414. doi: 10.3390/cancers15082304. PubMed PMID: 37190231; PubMed Central PMCID: PMCPMC10137288. |
| 33 | Ledenko 2023[33] | 33 | Ledenko M, Mercado L, Patel T. Predictors of Survival in Patients With Hepatocellular Cancer Receiving Atezolizumab and Bevacizumab. Am J Clin Oncol. 2024;47(3):105–9. Epub 20231204. doi: 10.1097/COC.0000000000001067. PubMed PMID: 38047447. |
| 34 | Song 2024[34] | 34 | Song BG, Goh MJ, Kang W, Sinn DH, Gwak GY, Choi MS, et al. Analysis of Factors Predicting the Real-World Efficacy of Atezolizumab and Bevacizumab in Patients with Advanced Hepatocellular Carcinoma. Gut Liver. 2024;18(4):709–18. Epub 20240627. doi: 10.5009/gnl240085. PubMed PMID: 38932499; PubMed Central PMCID: PMCPMC11249941. |
| 35 | Lee 2024[35] | 35 | Lee CL, Freeman M, Burak KW, Moffat GT, O'Donnell CDJ, Ding PQ, et al. Real-World Outcomes of Atezolizumab with Bevacizumab Treatment in Hepatocellular Carcinoma Patients: Effectiveness, Esophagogastroduodenoscopy Utilization and Bleeding Complications. Cancers (Basel). 2024;16(16). Epub 20240819. doi: 10.3390/cancers16162878. PubMed PMID: 39199649; PubMed Central PMCID: PMCPMC11352899. |
| 36 | Sho 2022[36] | 36 | Sho T, Suda G, Yamamoto Y, Furuya K, Baba M, Ogawa K, et al. Efficacy and Effect on Liver Functional Reserve of Atezolizumab and Bevacizumab for Unresectable Hepatocellular Carcinoma in Patients Who Do Not Meet Eligibility Criteria of IMbrave150. Cancers (Basel). 2022;14(16). Epub 20220815. doi: 10.3390/cancers14163938. PubMed PMID: 36010930; PubMed Central PMCID: PMCPMC9405784. |
| 37 | Nakagawa 2022[37] | 37 | Nakagawa M, Inoue M, Ogasawara S, Maruta S, Okubo T, Itokawa N, et al. Clinical effects and emerging issues of atezolizumab plus bevacizumab in patients with advanced hepatocellular carcinoma from Japanese real-world practice. Cancer. 2023;129(4):590–9. Epub 20221124. doi: 10.1002/cncr.34559. PubMed PMID: 36426410. |
| 38 | Singh 2025[39] | 39 | Singh SP, Kumar K, Kulkarni A, Arora V, Choudhury A, Chaubal A, et al. Predictors of Non-response to Atezolizumab Plus Bevacizumab in Patients With Unresectable Hepatocellular Carcinoma: A Multicentre Real World Study (HCC-AB Study). Journal of Clinical and Experimental Hepatology. 2025;15(4):102513. doi: 10.1016/j.jceh.2025.102513. PubMed PMID: 40129631. |
| 39 | Niizeki 2022[40] | 40 | Niizeki T, Tokunaga T, Takami Y, Wada Y, Harada M, Shibata M, et al. Comparison of Efficacy and Safety of Atezolizumab Plus Bevacizumab and Lenvatinib as First-Line Therapy for Unresectable Hepatocellular Carcinoma: A Propensity Score Matching Analysis. Target Oncol. 2022;17(6):643–53. Epub 20221022. doi: 10.1007/s11523-022-00921-x. PubMed PMID: 36272060; PubMed Central PMCID: PMCPMC9684293. |
| 40 | D'alessio 2022[41] | 41 | D'Alessio A, Fulgenzi CAM, Nishida N, Schonlein M, von Felden J, Schulze K, et al. Preliminary evidence of safety and tolerability of atezolizumab plus bevacizumab in patients with hepatocellular carcinoma and Child-Pugh A and B cirrhosis: A real-world study. Hepatology. 2022;76(4):1000–12. Epub 20220408. doi: 10.1002/hep.32468. PubMed PMID: 35313048; PubMed Central PMCID: PMCPMC9790703. |
| 41 | Takeuchi 2023[42] | 42 | Takeuchi Y, Nouso K, Fujioka SI, Kariyama K, Kobashi H, Uematsu S, et al. The prediction of early progressive disease in patients with hepatocellular carcinoma receiving atezolizumab plus bevacizumab. Cancer Med. 2023;12(17):17559–68. Epub 20230803. doi: 10.1002/cam4.6369. PubMed PMID: 37537956; PubMed Central PMCID: PMCPMC10523973. |
| 42 | Moriguchi 2024[43] | 43 | Moriguchi M, Okuda K, Horiguchi G, Kataoka S, Seko Y, Yamaguchi K, et al. Safety/efficacy of atezolizumab+ bevacizumab during anti‐platelet/anticoagulation therapy in unresectable hepatocellular carcinoma. Liver International. 2024;44(8):1751–61. doi: 10.1111/liv.15918. PubMed PMID: 38838097. |
| 43 | Kikuchi 2024[44] | 44 | Kikuchi T, Takeuchi Y, Nouso K, Kariyama K, Kuwaki K, Toshimori J, et al. Predictive factors for transition to conversion therapy in hepatocellular carcinoma using atezolizumab plus bevacizumab. Liver Int. 2024;44(6):1456–63. Epub 20240315. doi: 10.1111/liv.15907. PubMed PMID: 38488749. |
| 44 | Vithayathil 2023[45] | 45 | Vithayathil M, D'Alessio A, Fulgenzi CAM, Nishida N, Schonlein M, von Felden J, et al. Impact of body mass index in patients receiving atezolizumab plus bevacizumab for hepatocellular carcinoma. Hepatol Int. 2023;17(4):904–14. Epub 20230401. doi: 10.1007/s12072-023-10491-3. PubMed PMID: 37005953; PubMed Central PMCID: PMCPMC10386929. |
| 45 | Castro 2024[46] | 46 | de Castro T, Welland S, Jochheim L, Leyh C, Shmanko K, Finkelmeier F, et al. Atezolizumab/bevacizumab and lenvatinib for hepatocellular carcinoma: A comparative analysis in a European real-world cohort. Hepatol Commun. 2024;8(11). Epub 20241104. doi: 10.1097/HC9.0000000000000562. PubMed PMID: 39495153; PubMed Central PMCID: PMCPMC11537570. |
| 46 | Kaneko 2024[47] | 47 | Kaneko S, Asahina Y, Murakawa M, Ueyama S, Maeyashiki C, Watanabe H, et al. Prognostic significance of C-reactive protein in unresectable hepatocellular carcinoma treated with atezolizumab and bevacizumab. Hepatol Res. 2024;54(6):562–74. Epub 20240111. doi: 10.1111/hepr.14001. PubMed PMID: 38133587. |
| 47 | Kim 2024[48] | 48 | Kim DH, Min EJ, Kim B, Choi JY, Jang JW, Sung PS, et al. RECIST 1.1, mRECIST, and Choi criteria for evaluating treatment response and survival outcomes in hepatocellular carcinoma patients treated with atezolizumab plus bevacizumab. Eur Radiol. 2025;35(2):684–94. Epub 20240730. doi: 10.1007/s00330-024-10986-z. PubMed PMID: 39080067. |
| 48 | Ueno 2024[49] | 49 | Ueno M, Takeda H, Takai A, Morimura H, Nishijima N, Iwamoto S, et al. CRAFITY score as a predictive marker for refractoriness to atezolizumab plus bevacizumab therapy in hepatocellular carcinoma: a multicenter retrospective study. J Gastroenterol. 2024;59(12):1107–18. Epub 20240918. doi: 10.1007/s00535-024-02150-7. PubMed PMID: 39289234; PubMed Central PMCID: PMCPMC11541291. |
| 49 | Kim 2025[50] | 50 | Kim HD, Park YG, Hong H, Chung SW, Kim S, Ryu MH, et al. Atezolizumab plus Bevacizumab Is Associated with Favorable Overall Survival over Lenvatinib in Patients with Unresectable Hepatocellular Carcinoma. Oncology. 2026;104(1):40–50. Epub 20250321. doi: 10.1159/000545351. PubMed PMID: 40122043. |
| 50 | Cosgrove 2025[51] | 51 | Cosgrove D, Tan R, Osterland AJ, Hernandez S, Ogale S, Mahrus S, et al. Atezolizumab Plus Bevacizumab in Patients with Unresectable Hepatocellular Carcinoma: Real-World Experience From a US Community Oncology Network. J Hepatocell Carcinoma. 2025;12:791–804. Epub 20250418. doi: 10.2147/JHC.S492881. PubMed PMID: 40271535; PubMed Central PMCID: PMCPMC12015733. |
| 51 | Lee 2025[52] | 52 | Lee J, Yang K, Han JW, Sung PS, Jang JW, Yoon SK, et al. Subset of Child-Pugh Score 7 Shows Comparable Survival Outcomes to Child-Pugh Score 6 in Patients with Hepatocellular Carcinoma Treated with Atezolizumab and Bevacizumab. Clin Cancer Res. 2025;31(20):4323–32. doi: 10.1158/1078-0432.CCR-25-0495. PubMed PMID: 40407727; PubMed Central PMCID: PMCPMC12521912. |
| 52 | Kaplan 2024[53] | 53 | Kaplan DE, Tan R, Xiang C, Mu F, Hernandez S, Ogale S, et al. Overall Survival in Real-World Patients with Unresectable Hepatocellular Carcinoma Receiving Atezolizumab Plus Bevacizumab Versus Sorafenib or Lenvatinib as First-Line Therapy: Findings from the National Veterans Health Administration Database. Cancers (Basel). 2024;16(20). Epub 20241017. doi: 10.3390/cancers16203508. PubMed PMID: 39456602; PubMed Central PMCID: PMCPMC11506031. |
| 53 | Tada 2024[54] | 54 | Tada T, Kumada T, Hiraoka A, Hirooka M, Kariyama K, Tani J, et al. Outcomes of patients with hepatocellular carcinoma treated with atezolizumab plus bevacizumab in real-world clinical practice who met or did not meet the inclusion criteria for the phase 3 IMbrave150 trial. Aliment Pharmacol Ther. 2024;60(2):233–45. Epub 20240508. doi: 10.1111/apt.18037. PubMed PMID: 38716823. |
| 54 | Abdelrahim 2025[55] | 55 | Abdelrahim M, Esmail A, Kim RD, Arora SP, Arshad J, Kournoutas IA, et al. Real-World Experiences Using Atezolizumab + Bevacizumab for the Treatment of Unresectable Hepatocellular Carcinoma: A Multicenter Study. Cancers (Basel). 2025;17(11). Epub 20250529. doi: 10.3390/cancers17111814. PubMed PMID: 40507295; PubMed Central PMCID: PMCPMC12153728. |
| 55 | Allaire 2025[56] | 56 | Allaire M, Thiam EM, Amaddeo G, Bouattour M, Edeline J, Brusset B, et al. Real-World Outcomes of Atezolizumab-Bevacizumab in Hepatocellular Carcinoma: The Prospective French CHIEF Cohort. Liver Int. 2025;45(10):e70337. doi: 10.1111/liv.70337. PubMed PMID: 40905621; PubMed Central PMCID: PMCPMC12410122. |
| 56 | Ohara 2025[57] | 57 | Ohara M, Suda G, Kohya R, Yasui Y, Tsuchiya K, Kurosaki M, et al. Three-year overall survival in unresectable hepatocellular carcinoma treated with atezolizumab plus bevacizumab. Hepatol Int. 2025;19(6):1371–81. Epub 20250828. doi: 10.1007/s12072-025-10875-7. PubMed PMID: 40875102. |
| 57 | Chamseddine 2024[58] | 58 | Chamseddine S, LaPelusa M, Xiao L, Mohamed YI, Lee SS, Hu ZI, et al. Plasma Growth Hormone as a Prognostic Biomarker to Durvalumab and Tremelimumab in Patients with Advanced Hepatocellular Carcinoma. J Hepatocell Carcinoma. 2024;11:455–61. Epub 20240304. doi: 10.2147/JHC.S452564. PubMed PMID: 38463542; PubMed Central PMCID: PMCPMC10921889. |
| 58 | Kawamura 2025[60] | 60 | Kawamura Y, Akuta N, Shindoh J, Matsumura M, Okubo S, Tominaga L, et al. Peripheral blood CD4+/CD8+ T cell ratio may have potential for predicting the treatment response of durvalumab plus tremelimumab therapy (STRIDE) for unresectable hepatocellular carcinoma: Preliminary report. Hepatol Res. 2025;55(6):948–55. Epub 20250329. doi: 10.1111/hepr.14188. PubMed PMID: 40317865. |
| 59 | Fujii 2025[61] | 61 | Fujii Y, Kawaoka T, Shirane Y, Miura R, Nakahara H, Yamaoka K, et al. First-Line Durvalumab plus Tremelimumab Treatment for Unresectable Hepatocellular Carcinoma in Real-World Clinical Practice. Oncology. 2025;103(8):742–7. Epub 20241111. doi: 10.1159/000542517. PubMed PMID: 39527933; PubMed Central PMCID: PMCPMC12324697. |
| 60 | Fujiwara 2024[62] | 62 | Fujiwara Y, Kuroda H, Abe T, Kakisaka K, Nakaya I, Ito A, et al. Early Clinical Outcomes of Durvalumab Plus Tremelimumab in Unresectable Hepatocellular Carcinoma: A Real-World Comparison with First-Line or Later-Line Treatment. Drugs Real World Outcomes. 2024;11(4):701–10. Epub 20241010. doi: 10.1007/s40801-024-00458-7. PubMed PMID: 39384684; PubMed Central PMCID: PMCPMC11589085. |
| 61 | Honma 2025[63] | 63 | Honma Y, Shibata M, Ikemi M, Yoshitomi K, Shinohara N, Ogino N, et al. Usefulness of the Early Increase of Peripheral Blood Lymphocyte Count in Predicting Clinical Outcomes for Patients with Advanced Hepatocellular Carcinoma Treated with Durvalumab Plus Tremelimumab. Cancers. 2025;17(8):1274. doi: 10.3390/cancers17081274. PubMed PMID: 40282450. |
| 62 | Uchikawa 2024[64] | 64 | Uchikawa S, Kawaoka T, Murakami S, Miura R, Shirane Y, Johira Y, et al. Significance of changes in tumor markers in patients treated with durvalumab plus tremelimumab combination therapy as a surrogate marker for tumor response to unresectable hepatocellular carcinoma. Hepatol Res. 2025;55(1):149–54. Epub 20240817. doi: 10.1111/hepr.14104. PubMed PMID: 39152708. |
| 63 | Kuzuya 2024[65] | 65 | Kuzuya T, Kawabe N, Muto H, Wada Y, Komura G, Nakano T, et al. Early Changes in Alpha-Fetoprotein and Des-gamma-Carboxy Prothrombin Are Useful Predictors of Antitumor Response to Durvalumab Plus Tremelimumab Therapy for Advanced Hepatocellular Carcinoma. Curr Oncol. 2024;31(8):4225–40. Epub 20240726. doi: 10.3390/curroncol31080315. PubMed PMID: 39195298; PubMed Central PMCID: PMCPMC11353012. |
| 64 | Suzuki 2025[66] | 66 | Suzuki T, Matsuura K, Sobue S, Kato D, Hayashi K, Kondo H, et al. Neutrophil‐to‐lymphocyte ratio at the start of the second course of durvalumab plus tremelimumab therapy predicts therapeutic efficacy in patients with advanced hepatocellular carcinoma: A multicenter analysis. Hepatology Research. 2025;55(6):883–95. doi: 10.1111/hepr.14189. PubMed PMID: 40317623. |
| 65 | Sakane 2026[68] | 68 | Sakane T, Tada T, Tei H, Kushida S, Momose K, Hirano H, et al. Lymphocyte–Monocyte Ratio Predicts Survival in Patients With Unresectable Hepatocellular Carcinoma Treated With Durvalumab Plus Tremelimumab: A Multicenter Analysis. Hepatol Res. 2026;56(4):527–37. doi: 10.1111/hepr.70077. PubMed PMID: 41251032. |
| 66 | Tomonari 2025[69] | 69 | Tomonari T, Shimose S, Saeki I, Tani J, Honma Y, Ito T, et al. A novel approach to evaluate the therapeutic efficacy of durvalumab and tremelimumab combination therapy in hepatocellular carcinoma. Hepatol Res. 2025;55(8):1184–92. Epub 20250528. doi: 10.1111/hepr.14212. PubMed PMID: 40433908. |
| 67 | Celsa 2025[70] | 70 | Celsa C, Pressiani T, Nishida N, Chamseddine SM, Arvind A, Li M, et al. Reproducible safety and efficacy of durvalumab with or without tremelimumab for hepatocellular carcinoma in clinical practice: results of the DT-real study. JHEP Rep. 2026;8(3):101685. Epub 2025. doi: 10.1016/j.jhepr.2025.101685. PubMed PMID: 41675370. |
| 68 | Matono 2025[71] | 71 | Matono T, Tada T, Kumada T, Hiraoka A, Hirooka M, Kariyama K, et al. Safety and Efficacy of Durvalumab Plus Tremelimumab in Older Individuals With Unresectable Hepatocellular Carcinoma: A Multicenter Analysis. Hepatology Research. 2025;55(11):1507–17. doi: 10.1111/hepr.70011. PubMed PMID: 40767262. |
| 69 | Mohri 2026[72] | 72 | Mohri K, Nagai H, Mukozu T, Matsui T, Nagumo H, Yoshimine N, et al. Clinical Utility of Tremelimumab/Durvalumab as First-line Treatment for Unresectable Hepatocellular Carcinoma: Serum Cytokine Profile Insights. Anticancer Res. 2026;46(3):1545–56. doi: 10.21873/anticanres.18049. PubMed PMID: 41760263. |
| 70 | Bansal 2026[73] | 73 | Bansal S, Amin P, Williamson C, Valerio SJ, Kaplan DE. Outcomes with Single Tremelimumab Regular Interval Durvalumab (STRIDE) for Unresectable Hepatocellular Carcinoma in the US Veterans Administration. Cancers (Basel). 2026;18(7):1085. doi: 10.3390/cancers18071085. PubMed PMID: 41976308. |
| 71 | Korphaisarn 2026[74] | 74 | Korphaisarn K, Wirasorn K, Tanasanvimon S, Thanasombunsukh K, Maneenil K, Thanestada J, et al. Efficacy and Safety of Durvalumab plus Tremelimumab for Unresectable Hepatocellular Carcinoma: A Real-World Multicenter Observational Study. Oncol Res. 2026;34(9). doi: 10.32604/or.2026.083206. PubMed PMID: 42630704. |
| 72 | Mıdık 2026[75] | 75 | Mıdık MM, Şahin G, Durukan BM, Kuş F, Haşimoğlu Gürün K, Ünal S, et al. First-Line Durvalumab–Tremelimumab in Advanced Hepatocellular Carcinoma: Real-World Data from Turkey. J Clin Med. 2026;15(15):5997. doi: 10.3390/jcm15155997. PubMed PMID: 42590101. |
| 73 | Haghnejad 2023[76] | 76 | Haghnejad V, Muller M, Blaise L, Gerolami R, Bouattour M, Assenat E, et al. Atezolizumab plus bevacizumab in advanced hepatocellular carcinoma after treatment failure with multikinase inhibitors. Dig Liver Dis. 2023;55(7):938–44. doi: 10.1016/j.dld.2023.03.005. PubMed PMID: 37019737. |
| 74 | Dung 2025[77] | 77 | Dung NT, Binh NH, Quang BV, Hien TT, Phuong PC, Thang VH. Real-World Survival Outcomes of Atezolizumab and Bevacizumab in Unresectable Hepatocellular Carcinoma Patients With Hepatitis B Virus Infection: A Hospital-Based Cohort Study. Cancer Control. 2025;32:10732748251405547. doi: 10.1177/10732748251405547. PubMed PMID: 41388811. |
| 75 | Phillips 2026[78] | 78 | Phillips R, Daniels H, Quinlan M, Suwaidan A, Maru K, Hancock J, et al. Real-world evidence for atezolizumab with bevacizumab for unresectable hepatocellular carcinoma: a multicentre UK Midlands cohort. BMC Cancer. 2026. doi: 10.1186/s12885-026-16387-3. PubMed PMID: 42477572. |
| 76 | Tovoli 2026[79] | 79 | Tovoli F, Iavarone M, Cabibbo G, Marra F, Vivaldi C, Dalbeni A, et al. Long-term outcomes of atezolizumab–bevacizumab in unresectable hepatocellular carcinoma: a real-world study. Hepatology. 2026;84(3):756–68. doi: 10.1097/HEP.0000000000001725. PubMed PMID: 41746328. |
| 77 | Inoue 2025 | 67 | Inoue Y, Yano Y, Kushida S, Hirohata S, Yoon S, Yasutomi E, et al. Higher Absolute Lymphocyte Counts and Lower Des-gamma-Carboxyprothrombin Levels After Treatment Initiation Are Associated With the Clinical Efficacy of Tremelimumab Plus Durvalumab Combination Therapy for Hepatocellular Carcinoma. JGH Open. 2025;9(2):e70123. Epub 20250220. doi: 10.1002/jgh3.70123. PubMed PMID: 39989844; PubMed Central PMCID: PMCPMC11842866. |
| 78 | Piscaglia 2025 | 38 | Piscaglia F, Masi G, Martinelli E, Cabibbo G, Di Maio M, Gasbarrini A, et al. Atezolizumab plus bevacizumab as first-line treatment of unresectable hepatocellular carcinoma: interim analysis results from the phase IIIb AMETHISTA trial. ESMO Open. 2025;10(2):104110. Epub 20250127. doi: 10.1016/j.esmoop.2024.104110. PubMed PMID: 39874903; PubMed Central PMCID: PMCPMC11799967. |
| 79 | Shao 2025 | 59 | Shao YY, Chen CT, Chuang CH, Su TH, Ho MC, Tseng TC, et al. Prompt initiation of durvalumab and tremelimumab for unresectable hepatocellular carcinoma in patients with chronic active hepatitis B: a phase 2 clinical trial. Br J Cancer. 2025;132(9):822–7. Epub 20250324. doi: 10.1038/s41416-025-02978-7. PubMed PMID: 40128285; PubMed Central PMCID: PMCPMC12041533. |
